# LSTM-driven drug design using SELFIES for target-focused de novo generation of HIV-1 protease inhibitor candidates for AIDS treatment

**DOI:** 10.1371/journal.pone.0303597

**Published:** 2024-06-21

**Authors:** M. Taleb Albrijawi, Reda Alhajj

**Affiliations:** 1 Department of Computer Engineering, Istanbul Medipol University, Istanbul, Turkey; 2 Department of Computer Science, University of Calgary, Alberta, Canada; 3 Department of Health Informatics, University of Southern Denmark, Odense, Denmark; Redesign Science, UNITED STATES

## Abstract

The battle against viral drug resistance highlights the need for innovative approaches to replace time-consuming and costly traditional methods. Deep generative models offer automation potential, especially in the fight against Human immunodeficiency virus (HIV), as they can synthesize diverse molecules effectively. In this paper, an application of an LSTM-based deep generative model named “LSTM-ProGen” is proposed to be tailored explicitly for the de novo design of drug candidate molecules that interact with a specific target protein (HIV-1 protease). LSTM-ProGen distinguishes itself by employing a long-short-term memory (LSTM) architecture, to generate novel molecules target specificity against the HIV-1 protease. Following a thorough training process involves fine-tuning LSTM-ProGen on a diverse range of compounds sourced from the ChEMBL database. The model was optimized to meet specific requirements, with multiple iterations to enhance its predictive capabilities and ensure it generates molecules that exhibit favorable target interactions. The training process encompasses an array of performance evaluation metrics, such as drug-likeness properties. Our evaluation includes extensive silico analysis using molecular docking and PCA-based visualization to explore the chemical space that the new molecules cover compared to those in the training set. These evaluations reveal that a subset of 12 de novo molecules generated by LSTM-ProGen exhibit a striking ability to interact with the target protein, rivaling or even surpassing the efficacy of native ligands. Extended versions with further refinement of LSTM-ProGen hold promise as versatile tools for designing efficacious and customized drug candidates tailored to specific targets, thus accelerating drug development and facilitating the discovery of new therapies for various diseases.

## 1 Introduction

Human immunodeficiency virus (HIV) is the insidious culprit behind acquired immunodeficiency syndrome (AIDS) [1]. It continues to pose a major global public health challenge, affecting millions of people worldwide [2]. Once this virus enters the host bloodstream, it attacks the CD4 immune cells, weakening the immune system and decreasing the body’s ability to fight other infectious diseases. According to the latest statistics published by “Joint United Nations Program on HIV/AIDS” [3], there were 38.4 million people living with HIV in 2021, 1.5 million people became newly infected with the virus, and tragically, 650,000 individuals lost their lives to AIDS-related illnesses. This virus presents a menace to the health and well-being, especially for those individuals living in poverty-stricken countries with limited resources, and a lack of access to prevention and treatments. HIV/AIDS spreads through diverse ways, including unprotected sexual intercourse, sharing needles or syringes, and mother-to-child transmission during pregnancy, delivery, or breastfeeding [4]. These modes of transmission continue to provide considerable hurdles to HIV prevention efforts. Despite the progress in expanding access to antiretroviral therapy, a staggering number of people living with HIV, estimated at 16.3 million, still do not have access to life-saving treatment [3]. This not only puts their health at risk but also contributes to the ongoing spread of the virus.

**Fig 1:**
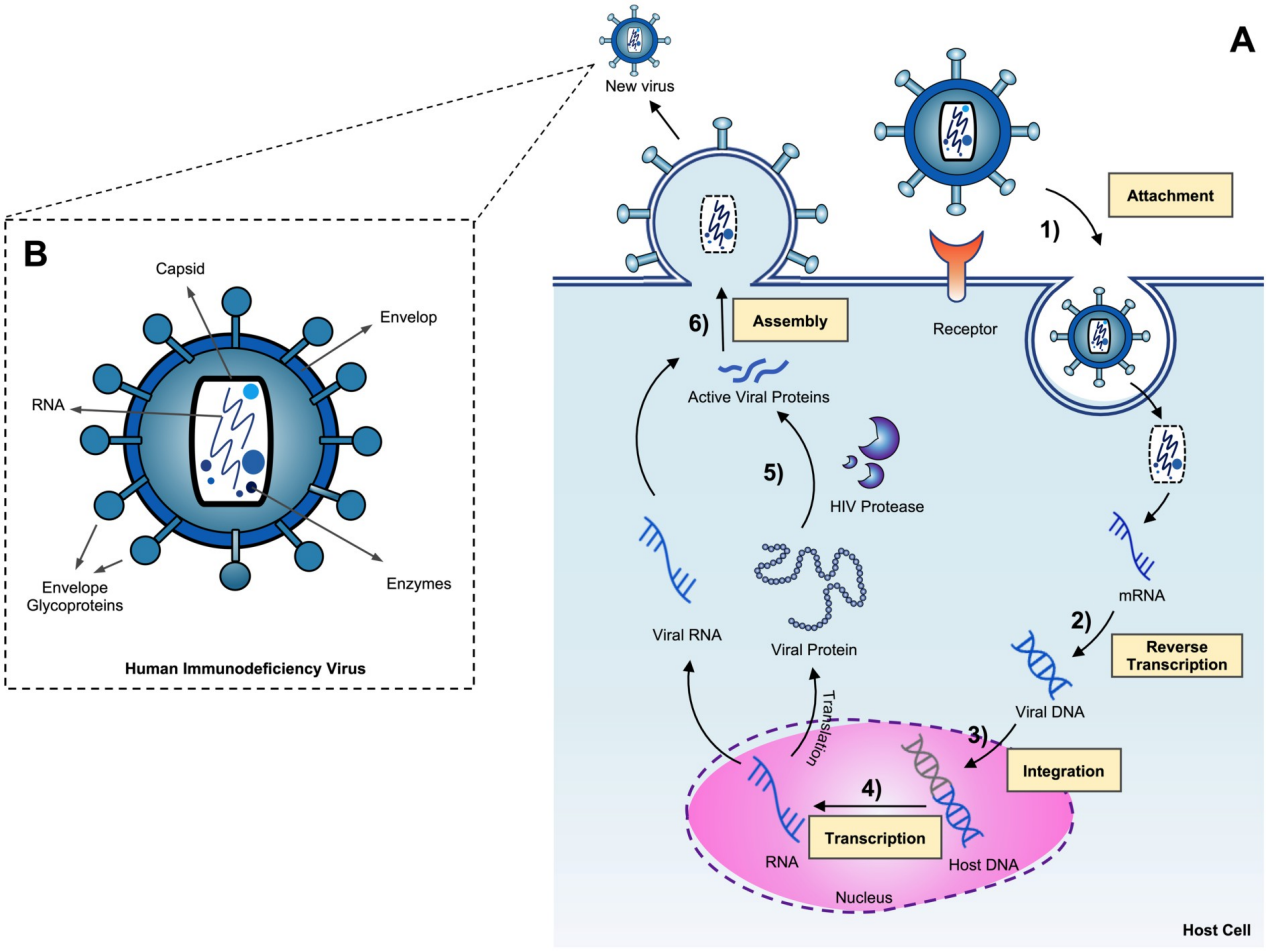
The lifecycle and structural composition of the HIV-1 virus. (A) The lifecycle of HIV-1 occurs in six major steps. 1) attaching and fusion of HIV virus, 2) reverse transcription, 3) integration of viral DNA into host DNA, 4) expression of viral genes, 5) protein cleavage process by HIV-1 PR, 6) viral assembly and produce new mature virion. (B) The virus contains two copies of the genetic material RNA and enzymes such as reverse transcriptase, integrase, and protease, which are crucial for its replication cycle, surrounded by a capsid protein shell. The capsid encased in a lipid membrane containing glycoprotein spikes.

The HIV-1 life cycle is complex, involving multiple steps that are essential for the virus to replicate and spread throughout the host’s body. It can be divided into six distinct stages [5], beginning with the attachment to the host cell membrane and fusion with a CD4+ T-lymphocyte Fig 1. Inside the cell, the viral RNA is reverse transcribed into viral DNA by the enzyme reverse transcriptase. Then, the viral DNA is transported to the host cell nucleus and integrates into the host chromosome, with the help of the integrase enzyme. Next, the virus uses the host’s cellular machinery to produce copies of its genomic material and creates messenger RNA strands, which are then translated into long chains of HIV-1 precursor proteins. These precursor proteins are then cleaved by the viral enzyme protease into smaller, active proteins that are then assembled into mature virions [6]. Once the viral RNA strands and active proteins are assembled into a new viral particle, the virus buds off from the host cell to infect another cell. The mature virion contains two copies of the viral genomic RNA and functional viral proteins, including reverse transcriptase, integrase, and protease.

**Fig 2:**
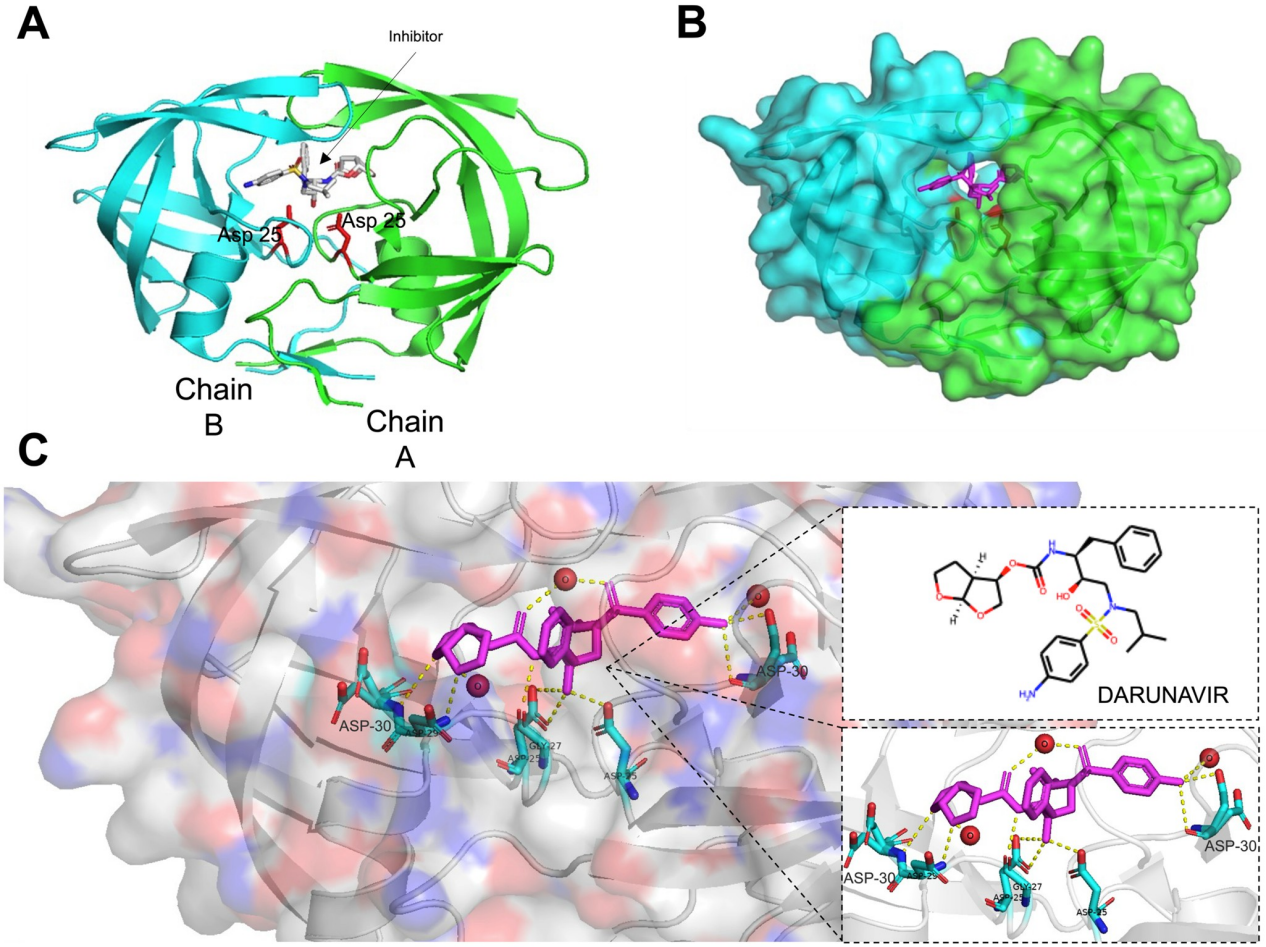
General overview of HIV-1 protease wild type in complex with Darunavir inhibitor. (A) Demonstrates the sec- ondary structure of HIV-1 protease, with active site residues highlighted in red. (B) The protein-ligand complex is represented in spheres with different colors for each chain. (C) Shows the protein-ligand interactions within the active site, with water molecules that interact with the ligand in the active site represented by the red spheres. PyMOL software was used to create these illustrations (PDB id “4LL3”).

As shown in Fig 1, the significance of HIV-1 protease in the virus’s life cycle is critical [1]; without its participation in cleaving viral polyproteins into functional active pieces, the process of reproducing new viruses will be halted. Hence, HIV-1 PR was a prime target for the development of antiviral drugs to treat HIV-1 infection [7]. The structure of HIV-1 protease is a dimer composed of two identical subunits, each of which contains 99 amino acid residues and a catalytic Asp at position 25 Fig 2.

**Fig 3:**
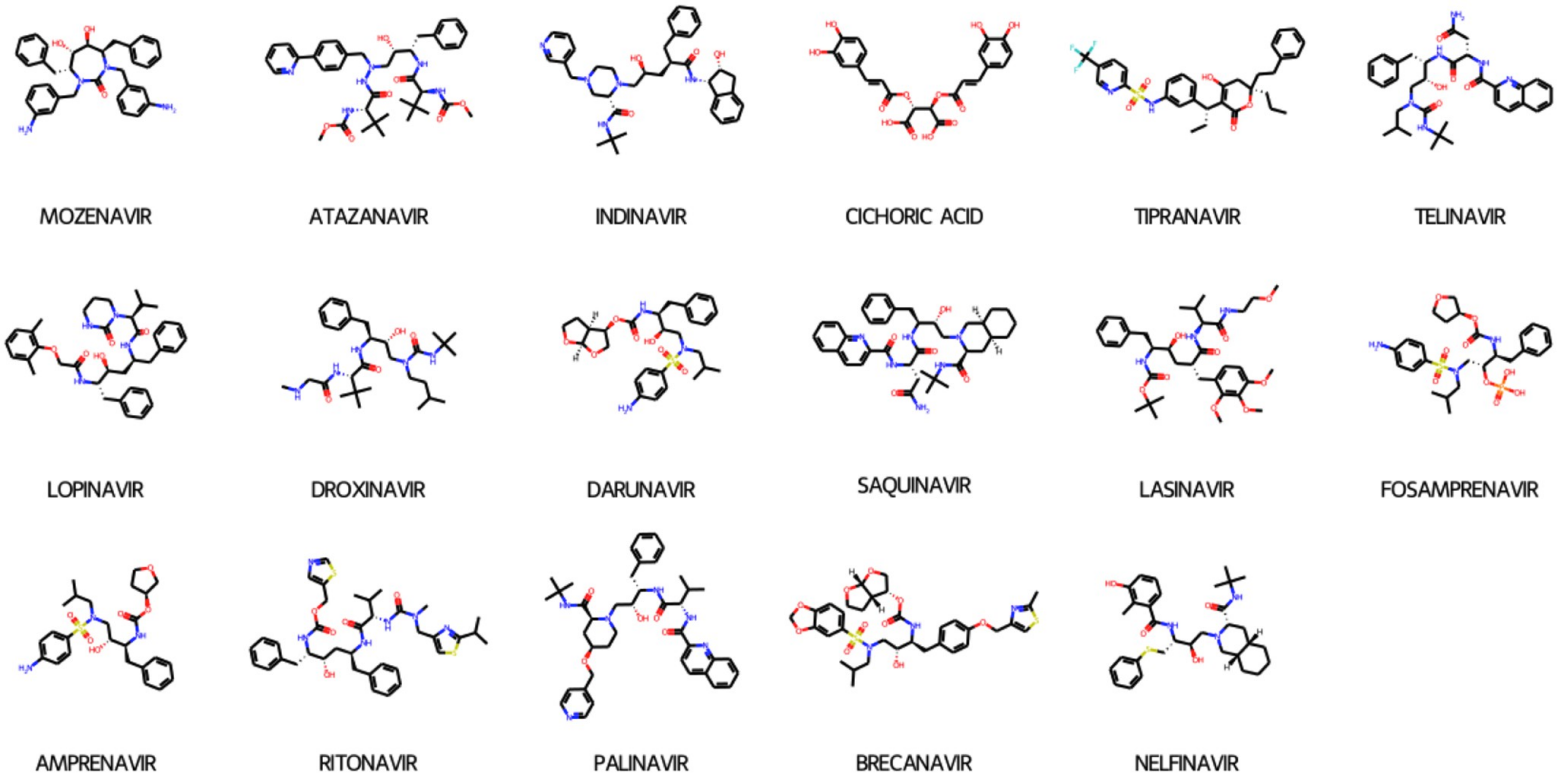
He known HIV-1 protease inhibitors. The structures of common HIV- 1 protease inhibitors. These inhibitors have been thoroughly researched and used in the treatment of HIV-related infections (AIDS).

Drugs that target the protease active site have been successful in treating HIV infection [8] Fig 3. However, these drugs are often associated with low efficacy due to the frequent emergence of protease structural mutations, which mostly can lead to drug resistance. Doctors occasionally combine medications to boost the efficacy of these drugs and control the progression of the disease depending on the patient’s conditions [9]. These facts emphasize the ongoing pressing need for finding new effective antiviral drugs, together with raising awareness and educating societies to prevent the spreading of the virus and support the efforts to save lives.

The regular process of bringing a new drug to the market requires serious investments of time, resources, and finance, as it is a complex and rigorous process of development, testing, and regulatory approval. It is estimated to average 10 years [10] with more than one billion dollars [11]. It starts with testing thousands of compounds to identify valuable drug candidates, then it goes through clinicals and most of the candidates fail. Researchers always try to develop innovative techniques to address the pressing need for new pharmaceuticals by deviating from standard procedures. With the recent advances in DL and ML approaches, existing drug and drug-like datasets, and the vast unknown chemical space [12], as well as the rise of molecular languages [13], it is now feasible to uncover novel drug candidates faster and at a lower cost.

In this study, we propose an innovative deep-learning model designed to generate novel candidate compounds specifically targeting HIV-1 protease as a potential therapeutic target for the treatment of AIDS. To achieve the goal, a meticulous multi-step process is employed, leveraging data from the ChEMBL database. The performance of the model is thoroughly evaluated using various metrics, ensuring a comprehensive analysis. The key contributions and noticeable achievements are listed below.

- Development of a multi-layered novel deep learning framework for generating new molecule candidates targeting HIV-1 protease.
- Implementation of an LSTM-based model trained within a pyramid scheme training strategy to bias the generation process towards the target protein.
- Utilization of the SELFIES molecular language for encoding and decoding molecules for feeding up the proposed model.
- Evaluation of the molecular modeling performance of the generated molecules using various methods inspired by RDkit, a widely used cheminformatics library.
- Conducting a molecular docking study to assess the binding affinity and potential interactions between the final filtered generated molecules and the HIV-1 protease target.
- Exploration of different strategies and techniques to enhance the generation of molecules with improved properties and potential for inhibiting the HIV-1 protease.
- Contribution to the field of computational drug discovery by proposing a deep learning-based approach for generating novel molecules targeting specific proteins involved in diseases with a novel training strategy.
- The potential for discovering new lead compounds for developing anti-HIV drugs through the generated molecule candidates.
- Enhancing the understanding of the capabilities and limitations of deep learning frameworks for generating molecules and their evaluation in the context of HIV-1 protease drug discovery.

## 2 Related work

The area of drug discovery research rapidly advances to meet the ongoing need for new, and effective medications. Deep learning generative models have shown untapped potential for the discovery of novel drug candidates along with the huge public chemical compound databases such as ChEMBL [14] and DrugBank [15], and the vast unexplored chemical space [12, 16]. The number of potential undiscovered chemical compounds has been estimated to be upwards of 10^6^ [12, 16]. This suggests that there might be millions of new molecules with unique chemical characteristics that could lead to the development of innovative and more effective medications. Recently, various deep-learning models have been proposed and applied to generate novel molecules with desirable properties. In this section, we review some promising proposed models for molecular generation and show how they have assisted in the drug development process. The unifying thread connecting all these models is the need to discover a means to steer the generation process after training to be focused on the target.

Gómez-Bombarelli et al. [17] used a variational autoencoder (VAE) based model in their approach for developing compounds with specified attributes such as high chemical stability and solubility. They trained the VAE model to understand the underlying distribution of chemical structures in the used dataset, allowing it to build unique compounds with desired characteristics. They proved the efficacy of their method on many datasets, including the QM9 database [18]. Their technique generates novel compounds with certain properties; however, it requires very large datasets to train the model on, which significantly increases the computing cost.

Jin et al. [19] have developed an innovative approach for generating molecular graphs, named junction tree variational autoencoder (JT-VAE). It utilizes the advantages of a graph-based representation of molecules, with nodes representing atoms and edges representing chemical bonds. The JT-VAE generates new, valid molecular structures with desired properties by iteratively expanding and linking groups of connected atoms. This strategy intriguingly shows a promising methodology in the drug development process. While the approach is effective in learning the latent distribution of molecular networks, it relies on an algorithm to convert these graphs into actual molecular structures, which may not always fulfill expectations and the decision on the gradually added fragments. Furthermore, the computational expense of transforming molecules into graphs and training two sub-models may present difficulties.

As previously stated, generative adversarial network (GAN) is also widely employed for molecular generation. For the same purpose, Prykhodko et al. developed a novel design termed LatentGAN [20], which is a truly unique deep generative model for de novo molecule generation that combines an RNN-based heteroencoder with a generative adversarial network with two sub-models: generator and discriminator. They first trained the model on a portion of the ChEMBL [21] database of drug-like molecules and then retrained it on three target-biased datasets to generate target-biased compounds. Following this procedure, their results demonstrated that LatentGAN is a powerful tool for creating novel biased compounds. However, there is little empirical data to support the validity and usefulness of these novel compounds, necessitating more research. Encoding molecules to latent vectors without a thorough investigation and the high computational cost involved in training multiple models separately raise concerns about the methodology’s efficacy. MolGAN [22] is another implicit generative model for producing small molecules, introduced by De Cao and Kipf. It is made up of two primary components, as is typical of GAN models: a generator network and a discriminator network. The generator network creates new molecular graphs, while the discriminator network verifies the resulting graphs’ validity. The two networks are trained in a competitive way. MolGAN is trained on the Zinc database, which contains over 250,000 compounds, and creates new molecular graphs comparable to those in the database. MolGAN was capable of creating high-quality graphs while still retaining features critical for drug discovery and development, such as solubility and drug-likeness.

RNN-based models’ success in natural language processing has inspired researchers to adapt this methodology to molecular science. These models have successfully generated new compounds with promising drug discovery potential by modeling molecules in linear presentation languages such as SMILES, SELFIES, and InChI. The combination of machine learning and molecular science throws up fascinating potential for the development of novel treatments, leading to a promising future research field. Segler et al. [23] is a potential step forward in the development of novel methodologies for de novo molecular design. They defined a three-layer RNN-LSTM model for developing customized libraries targeting the serotonin receptor as well as other antibacterial targets. They trained the model with SMILES molecules from the Zinc15 database. They refine the model with small groups of compounds known to be active against that target. The authors compared the chemical space that the new compounds cover to that of the molecules in the training set as a way to evaluate the new molecules. Additionally, another successful study proposed by Gupta et al. presented an LSTM-based model for generating molecular libraries with structural similarity to known actives for *PPAR*_*γ*_ and Trypsin. The model was trained using 550,000 SMILES strings of active chemicals from ChEMBL before being fine-tuned with SMILES strings of 4,367 *PPAR*_*γ*_ ligands and 1,490 Trypsin inhibitors. Around 90% of the created compounds are distinct from the known ligands and from one another. The suggested approach was also evaluated for fragment-based drug development. This, together with the other models we have discussed, highlights the extraordinary potential of new deep generative approaches in the domain of drug discovery. Following this extensive review of the most promising molecular generative models, it is important to highlight their limitations and identify weaknesses in them. By noting these shortcomings, we can open the doors for novel approaches that overcome the current challenges and contribute to the development of more effective approaches. In the following sections, a list of three main notable shortcomings observed in the existing works is included below, focusing on places where changes are needed. By acknowledging these shortcomings, we can lay the groundwork for innovative solutions and methodologies to advance in this field.

In existing research, commonly used techniques heavily rely on graph representations to encode molecules, benefiting from graph theory’s advantages. However, our research acknowledges the computational expense associated with several graph-based deep learning methods for molecular graph generation, as these methods often demand substantial computational resources due to their holistic approach to processing entire graph structures. In contrast, our proposed method offers an alternative that is computationally less burdensome, leveraging LSTM models operating on sequential SELFIES strings. Graph-based models entail processing complex molecular graph structures, which inherently require handling diverse topologies, variable-sized graphs, and higher-dimensional data, given their need to capture intricate structural features such as cycles, branches, and functional groups. Moreover, the sequential generation process employed by graph-based models involves making sophisticated decisions at each step, further contributing to the computational overhead. The “Junction Tree Variational Autoencoder for Molecular Graph Generation” approach illustrates the computational intricacy of graph-based methods, requiring an autoencoder network comprising encoder and decoder networks, alongside tasks such as encoding molecular graphs to a latent space and decoding them back from the latent space. In contrast, our proposed model, utilizing LSTM networks on sequential SELFIES representations, offers a computationally more efficient alternative. LSTM models predict one atom at a time, generating molecules in a sequential manner, thereby simplifying the computational process compared to the holistic approach of graph-based methods, such as MolGAN, which involves training multiple components, including generators, discriminators, and reward networks, collectively contributing to heightened computational demands.

In conclusion, our method presents a computationally less expensive alternative to existing graph-based approaches for molecular graph generation. By leveraging LSTM models operating on sequential SELFIES strings, we mitigate the computational burden associated with processing entire graph structures, providing a more efficient solution for molecular generation tasks.

## 3 Methodology

### 3.1 Data collection

**Fig 4:**
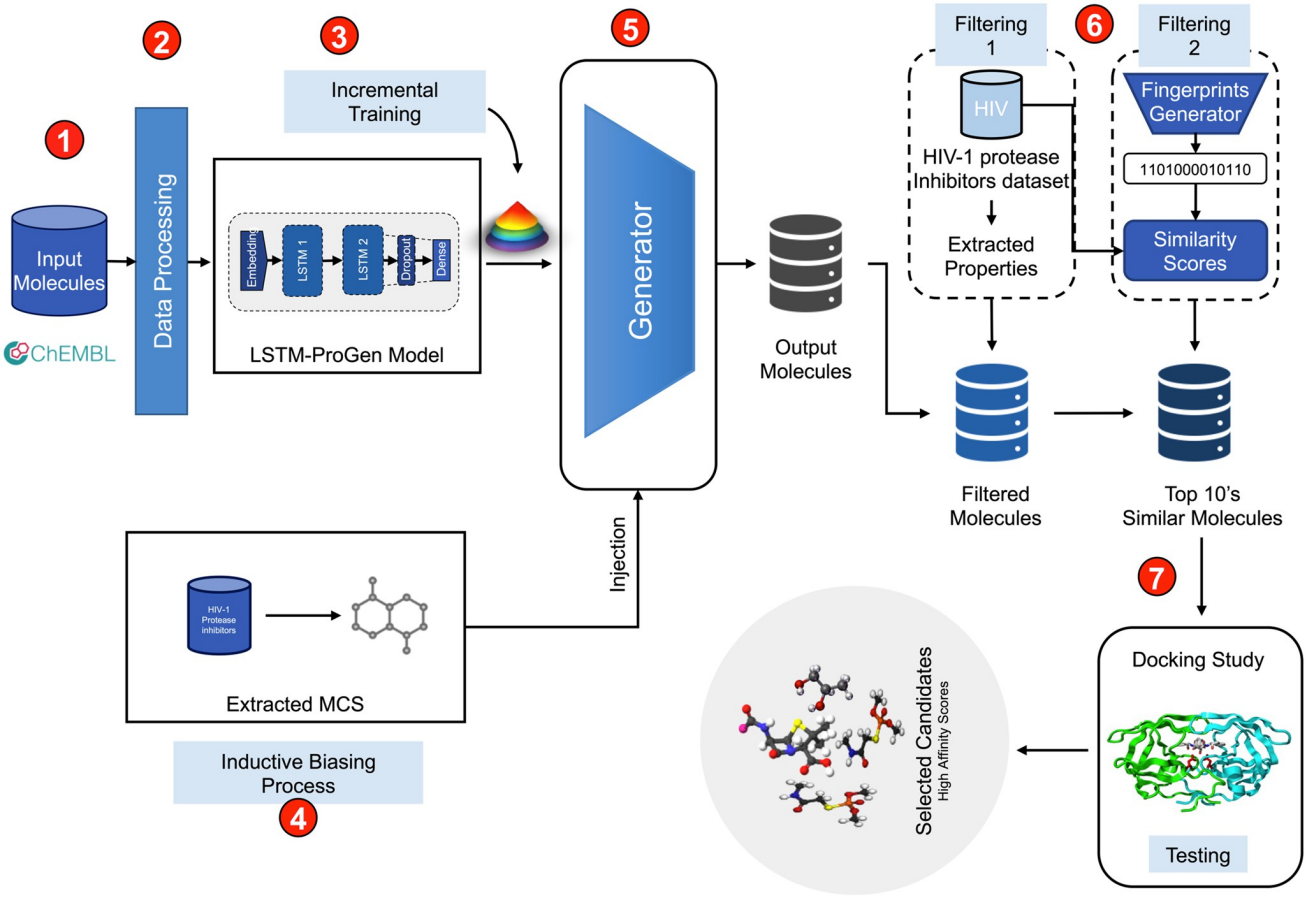
An overview of the proposed multi-step approach.

In the study, several datasets from the ChEMBL database [14, 21] were utilized for the investigation to accomplish the objective. ChEMBL database is an extensive collection and open-access database. It contains bioactive compounds with drug-like qualities and offers details on their biological functions, pharmacological characteristics, and molecular structures. The initial version of ChEMBL was created and maintained by researchers at the European Bioinformatics Institute (EBI). The ChEMBL project was established to fill the demand at the time for a centralized repository of bioactivity data. The overall methodology of the proposed approach is summarized in Fig 4, which provides an overview of the multi-step process employed in this study. The data was collected from a variety of sources throughout the years, including books, patents, and open databases. The database now has more than 2 million bioactivity data points, including more than 1.8 million chemicals and 15,000 targets. The database encourages researchers to share data and work together, which can speed up drug development studies and deepen our understanding of the underlying causes of disease. It served as a helpful resource for drug discovery research, especially for small and medium-sized businesses, university researchers, and non-profit groups that lacked access to private databases.

The first used dataset in our study is “14K drug” the sub-dataset from the ChEMBL database. It contains around 14,000 authorized small molecules with known drug-like properties such as the molecular structure, molecular weight, number of hydrogen bond acceptors and donors, partition coefficient, and other physicochemical parameters are all contained in this extensive dataset. The primary objective of utilizing this dataset was to enhance the model’s ability to generate compounds with drug-like features similar to those found in the “14K drugs” dataset. By training the model on this reliable molecule bank, we aimed to ensure that it learns and reproduces drug-like properties effectively. We applied filters to remove only the unfavorable molecules that did not share characteristics with well-known HIV-1 protease inhibitors. The resulting dataset was 9480 molecules with their characteristics. The main goal of utilizing this dataset was to improve the model’s capacity to produce compounds with drug-like features similar to those in this dataset.

Hepatitis C virus (HCV), Equine Infectious Anemia Virus (EIAV), West Nile virus (WNV), and SARS-CoV-2 protease inhibitors with a comparable mechanism to the HIV-1 protease were the only ones left after filtering the “14K Drugs” dataset to create the second dataset. After cleaning this dataset, it only contained 391 molecules with properties like molecular weight, octanol-water partition coefficient (LogP), Polar surface area, and others. With the help of this strategy, the model was able to concentrate on the target as a whole and generate compounds that were comparable to those in the training set, starting with drug-like molecules and moving toward protease inhibitors.

The approved HIV-1 protease inhibitors dataset, which includes 17 compounds, was the final dataset utilized in this study. It was used lastly to train the model to produce similar compounds with drug-like and HIV-1 protease inhibitor characteristics.

### 3.2 Exploring HIV-1 protease inhibitors dataset

**Fig 5:**
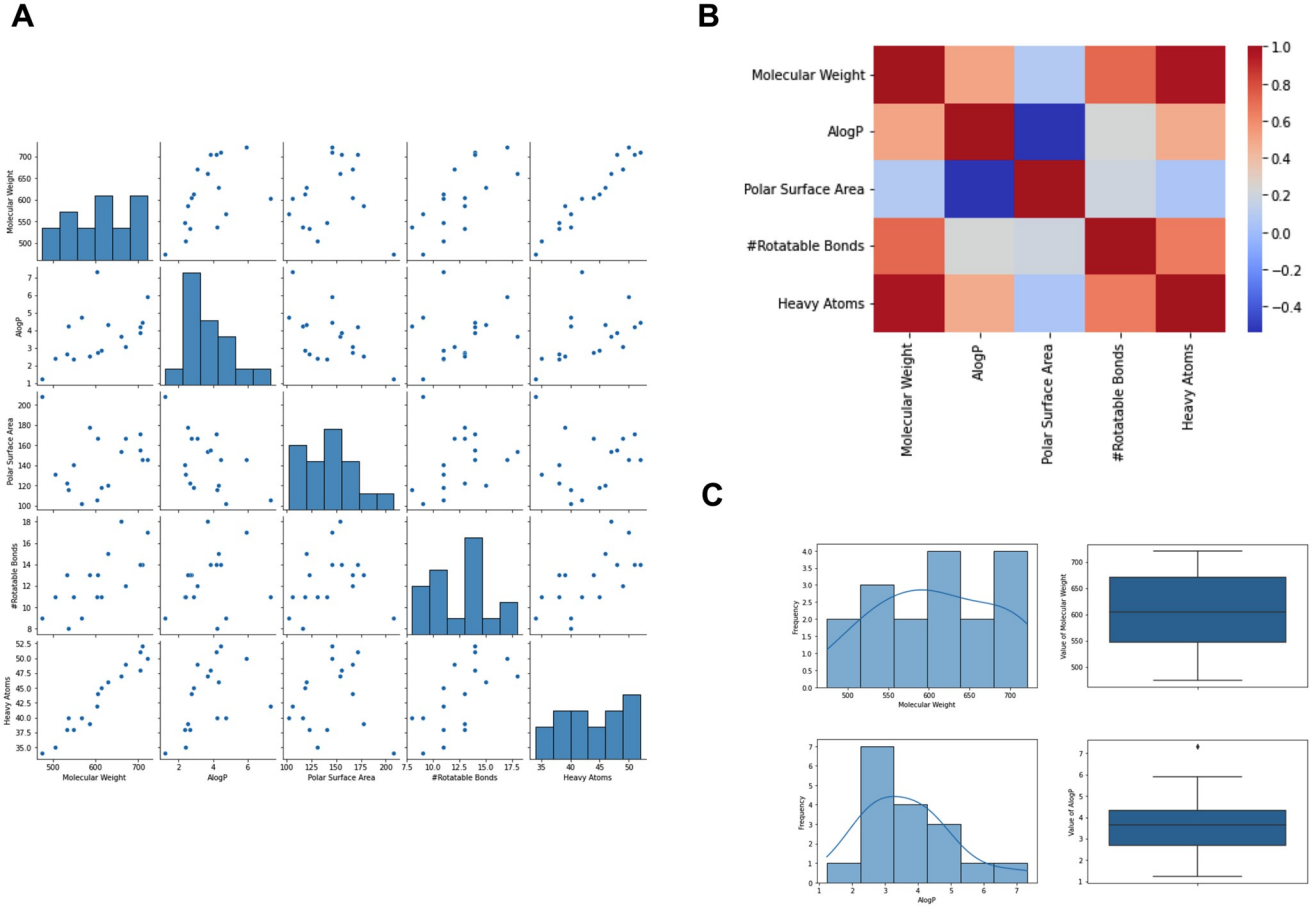
Examining the dataset of HIV-1 protease inhibitors. (A) Display the interactions between certain variables in the dataset. (B) The heatmap displays the correlations between the dataset attributes. (C) Histograms and boxplots display the molecular weight and AlogP ranges in the dataset.

We analyzed the dataset of known HIV-1 inhibitors to gain a deep understanding of the relationships between certain features and ensure the efficacy of the newly synthesized compounds against HIV-1 protease. As shown in Fig 5, we created a heatmap to show the connections between different metrics. The range of values for the selected molecular features, such as the number of rotatable bonds and polar surface area, was detected and listed in Table 1. With this knowledge, we can filter the newly created compounds based on these precise ranges of values for the given attributes. This will considerably increase their capacity to target and block HIV-1 protease. Using this method, we can dramatically boost the chance of discovering good candidates for future drug development efforts.

**Table 1:** Examining the distribution of the molecular properties of the HIV-1 protease known inhibitors dataset.

Measure	MW	AlogP	PSA	RB	HA
Mean	610.048	3.685	143.927	12.529	43.411
Std	76.350	95.86	95.86	97.42	97.42
Min	474.37	1.470	28.669	2.718	5.657
25%	547.67	95.41	94.87	97.17	97.15
50	604.75	1.23	101.9	8	34
75%	670.86	93.33	95.49	95.64	96.01
Max	720.96	2.68	120	11	39

MW is molecular weight, AlogP is the partition coefficient of a solute between octanol and water, PSA is the polar surface area, RB is the rotatable Bonds, and HA is the heavy Atoms.

### 3.3 Data augmentation

The limited number of data samples in the last two datasets was one of the main obstacles we faced in this study. Training our model with a low number of samples, particularly in the case of the HIV-1 protease inhibitors dataset, presented a tough challenge in producing similar samples of training examples to guarantee optimal model performance. To master the lack of data, we employed an innovative technique called “Smile Enumeration” to augment our data samples and increase the size of the datasets. It was previously published by E.J. Bjerrum [24] and has proven to be very promising for data augmentation and enhancing the performance of molecular models. The updated sizes of the last two used datasets are as follows: 2756, and 1717 for the second, and third datasets respectively.

This method, also known as SMILES enumeration, increases the dataset size by generating multiple unique SMILES representations for each molecule through random atom order changes. This method takes advantage of SMILEA representation of molecules as strings and one molecule can have multiple SMILES strings due to variations in atom ordering. By randomly changing the atom order of the molecule and generating new SMILES strings, unique representations of the same molecule are produced. For instance, applying SMILES enumeration to the molecule “CCC(=O)O[C@@]1(CCNH+C)c2ccccc2” produces three distinct SMILES:

- c1cccc([C@@]2(OC(CC)=O)CCNH+C[C@H]2CC = C)c1
- C([C@H]1C@@(c2ccccc2)CCNH+C1)C = C
- c1ccc([C@]2(OC(=O)CC)C@HCNH+CC2)cc1

By employing SMILES enumeration, the datasets size is significantly increased, leading to a more robust training dataset. This augmentation enhances the performance of molecular models by providing more diverse examples for training.

### 3.4 Maximum common sub-structures

**Fig 6:**
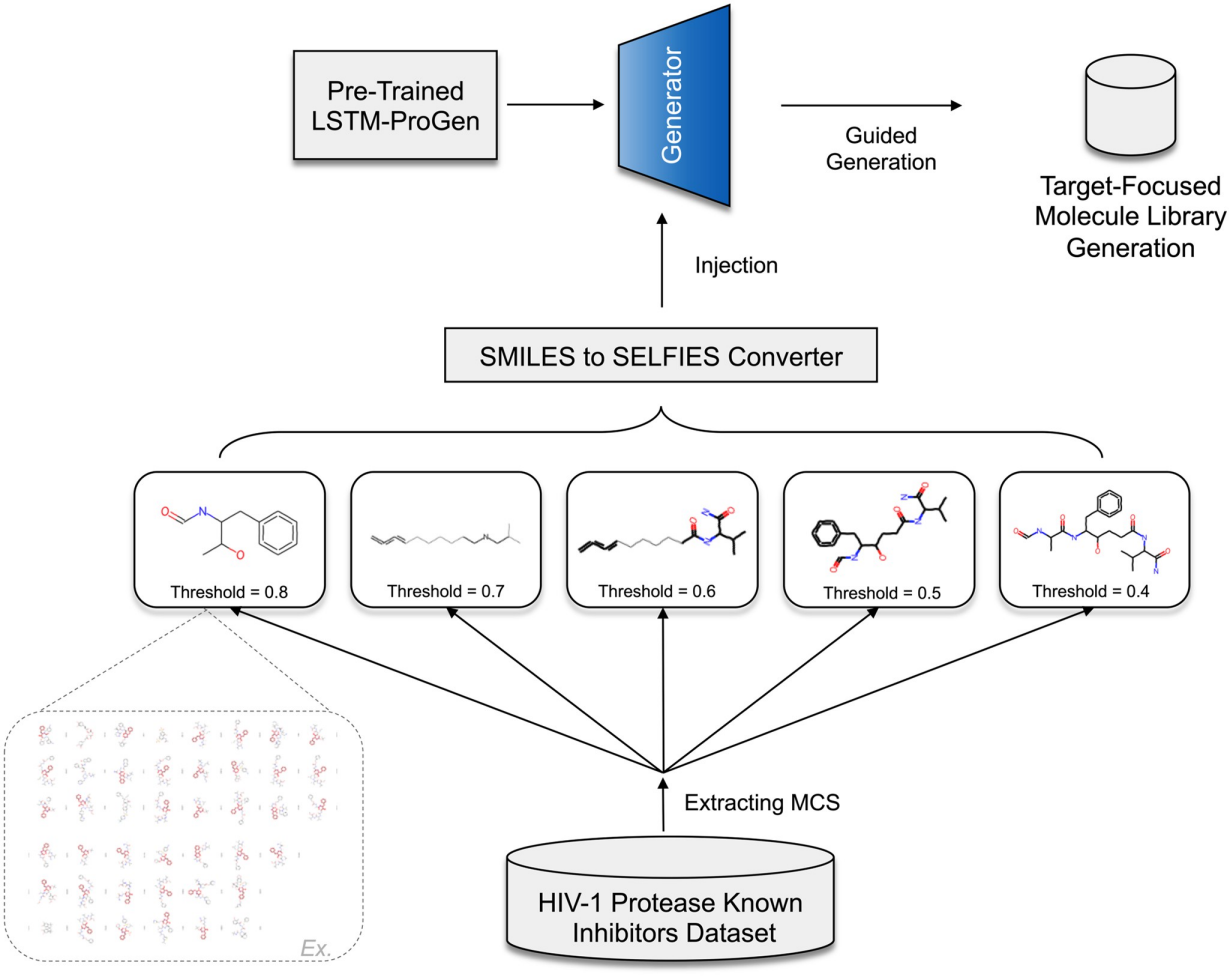
Queries of common substructures of known HIV-1 protease inhibitors. The process of extracting the most common substructures (MCS) from a dataset of known HIV-1 protease inhibitors at various thresholds obtained from the ChEMBL database, converting these substructures to SELFIES representation, and injecting them into the generator function to direct the generation process to be focused on the target.

As shown in Fig 6, the maximum common sub-structures (MCS) between the known HIV-1 protease inhibitors were extracted using a straightforward function from the RDkit library [25]. This function (rdkit.Chem.rdFMCS.FindMCS) takes a list of molecules and returns the MCS as a SMILES Arbitrary Target Specification (SMARTS) pattern. The extracted MCS will then be injected into the generator function to improve the newly generated compound’s ability to result in similar structures to the known HIV-1 protease inhibitors as we will show in the coming sections.

### 3.5 Data processing

**Fig 7:**
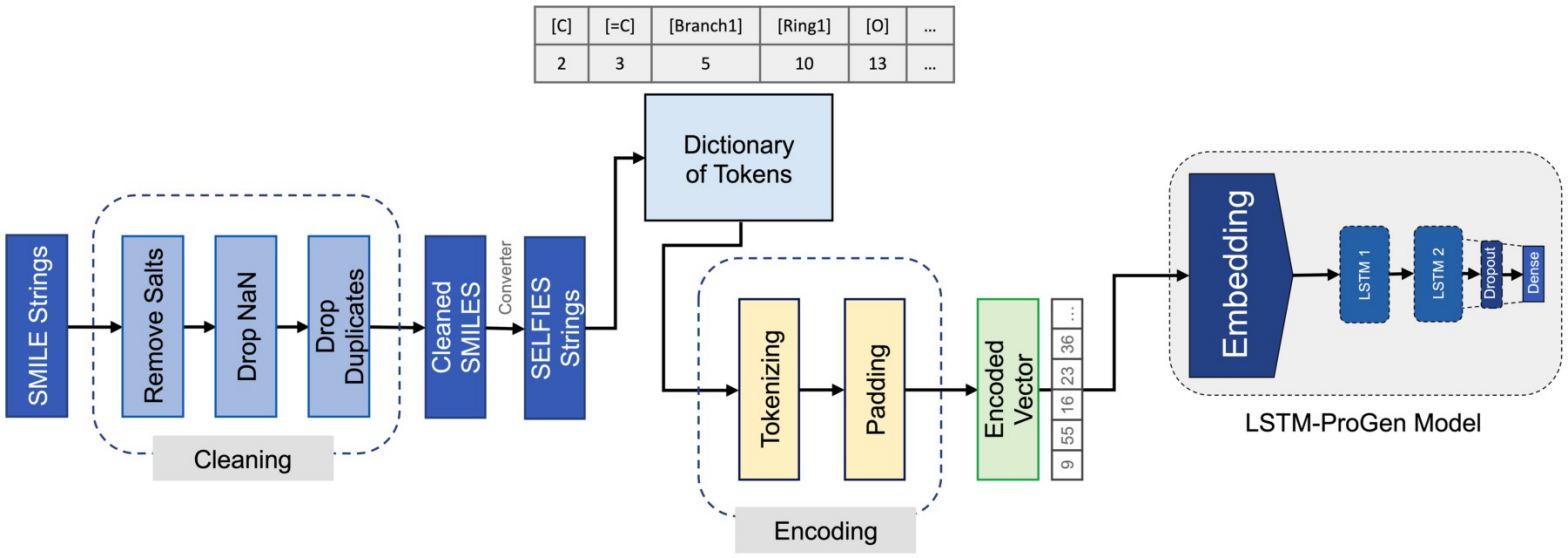
An overview of the data processing procedure. FData processing included cleaning the datasets from salts, NaNs, and duplicated rows; converting SMILES to SELFIES representations; creating a token dictionary that assigned a unique number to each character found in the datasets; encoding each molecule into a vector; and finally padding these vectors into a fixed length of 100 characters. The resulting vectors were fed into the defined model (LSTM-ProGen) at the first embedding layer.

Before starting the training process, the datasets must be ready to guarantee that the created models can learn effectively. As seen in Fig 7, this pretreatment process consists of several steps, including purging the datasets of various contaminants, including salts, NaNs, and duplicate rows. The next step is to convert the SMILES format to SELFIES representations once the datasets have been cleaned.

The primary input features utilized for characterizing the training data primarily revolve around the tokenized SELFIES representations of molecules. Each molecule in the dataset is represented as a sequence of tokens derived from its SELFIES string. Characterization in this context involves extracting relevant features from the SELFIES representations, such as the sequence of characters, bond types, and structural information embedded in the strings. By analyzing and processing these SELFIES strings, the LSTM model can learn the syntax and semantics of molecular structures to generate novel molecules that are chemically plausible. Therefore, the key input features for characterizing the training data in this scenario would be the SELFIES representations of molecules, which serve as the foundation for training the LSTM model to generate similar molecules based on learned patterns and structures.

**Fig 8:**
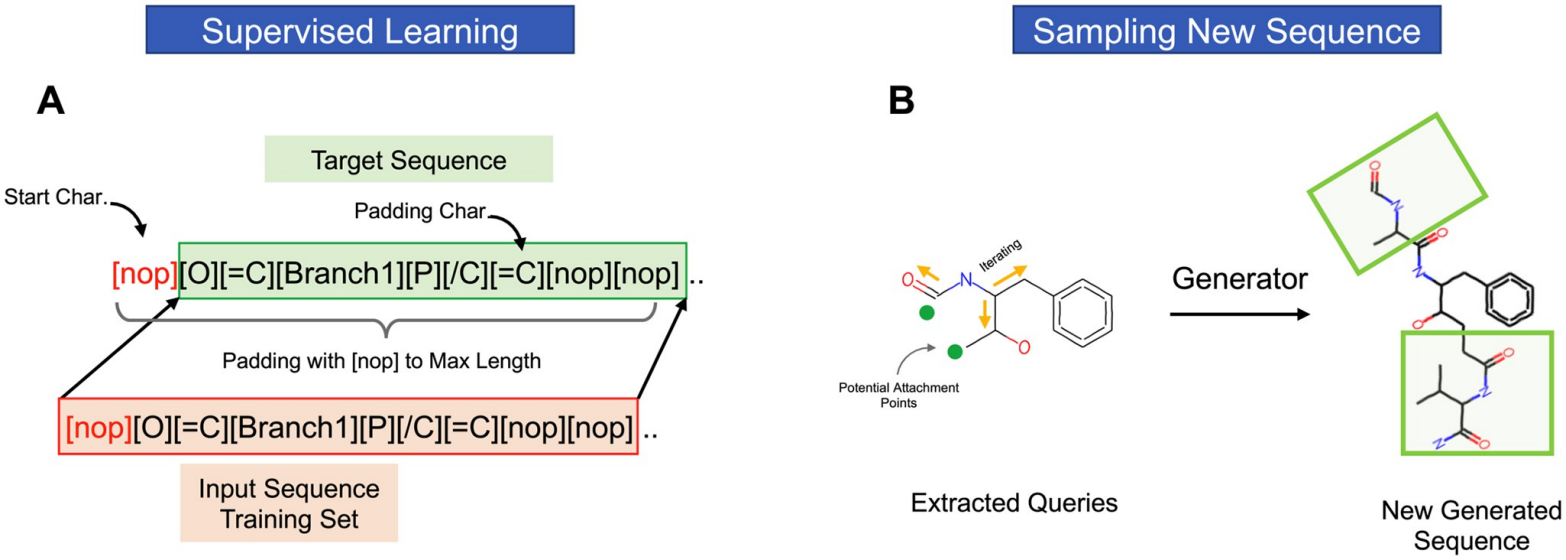
Molecular sequences preparation. Assigning labels to the sequences. (A) Each sequence had the prefix “[snop]” assigned to the beginning, and the length of each molecule was padded to the longest string found in the datasets. denoted by “[nop],”. As the model was being trained to learn it, the red sequence was initially used as the input and the green sequence as the goal. (B) Explains the sampling process of a new sequence using the trained model and the extracted queries, The generator function iterates through each query and adds new characters based on the information it learned from the training phase.

Later, the dataset was split randomly into two sets: a training set with 76% of the data and a test set with 24% of the data. We used a sliding window method that was proposed by Gupta et al. [26] Fig 8. Every sequence is prefixed with the character [snop] at the beginning of the sequence to differentiate between the training and testing sequences. Next, a token dictionary is created to give each unique character found in the datasets a special number. There were 86 of these characters. This enables us to vectorize the molecules and encode them for feeding into the model. Finally, each sequence was padded into a constant length of 100 characters, which is the maximum length of string found in the dataset, to ensure stability in the learning process of rules. The LSTM-ProGen model’s first embedding layer is then fed with the resulting encoded molecules.

### 3.6 LSTM-ProGen model

#### 3.6.1 Model architecture

**Fig 9:**
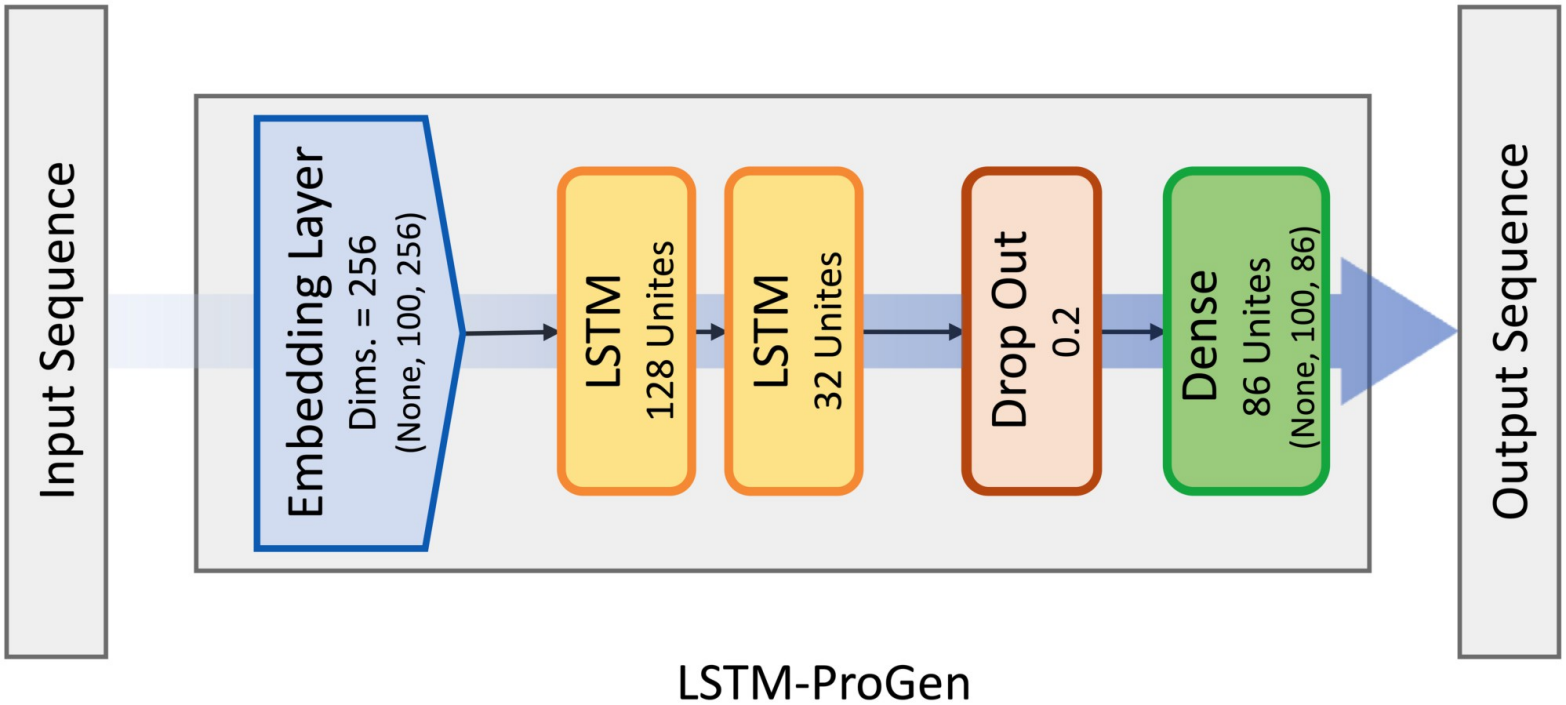
LSTM-ProGen model architecture. Illustration of the LSTM-ProGen architecture used for designing HIV antiviral drugs. Embedding layer, followed by 2 LSTM layers with 128 and 32 units respectively, Dropout layer and Dense layer with 86 units, and SoftMax activation function.

With the goal of creating new drug candidates for HIV/AIDS, we proposed two distinct models with slightly different processing of the input data. As shown in Fig 9, the proposed architecture consists of five layers: an embedding layer, followed by two LSTM layers, a dropout layer, and lastly a dense layer. The embedding layer transforms each character in the input sequence into a vector representation in a dense vector space that can depict the semantic meaning of the characters and their relationships. This enables easier the model to understand the given sequence and helps it to observe more useful characteristics. The model can learn to generalize more effectively by using an embedding layer, which makes it possible for it to recognize and understand similar characters or sequences of characters even when they weren’t in the training set of data [27]. The two LSTM layers have 128 and 32 hidden units, respectively, and allow the model to capture the temporal dependencies of the input sequence. The dropout layer helps prevent overfitting by randomly removing connections between the LSTM layers. The dense layer then converts the output of the LSTM layers into an output vector with 86 dimensions. Finally, the dense layer then converts the output of the last LSTM layers into an output vector with 86 dimensions. The total number of trainable parameters in this model is 242,582. The first LSTM layer has 197,120 parameters, the second LSTM layer has 20,608 parameters, and the dense layer has 2,838 parameters. The embedding layer has 22,016 parameters. This design was inspired by the natural language processing application and successfully balances computational efficiency and complexity, making it effective for a range of applications.

#### 3.6.2 Activation function

In our proposed model, we used SoftMax [28] as the activation function, which maps the output of the final dense layer to a probability distribution over the possible characters in the molecule vocabulary. The SoftMax formula is as follows:

$$f{\left(s\right)}_{i}=\frac{e^{s_{i}}}{\sum_{j=1}^{k}e^{s_{j}}}$$ (3.1)

In Eq (3.1), where *s* is the input vector made up of (*s*^0^, …, *s*^*k*^), all the *s*^*i*^ values are the elements of the input vector to the SoftMax function, $e^{s_{i}}$ is the standard exponential function applied to each element of the input vector, $\sum_{j=1}^{k}e^{s_{j}}$ is the normalization term, which assures that all the function’s output values add to 1 and are in the range (0, 1), resulting in a legitimate probability distribution, and *k* is the number of classes. SoftMax is particularly useful for multiclass classification problems because it ensures that the sum of the probabilities for all possible classes is equal to 1. This means that the model can generate new molecules that are valid and follow the rules of the chemical language.

#### 3.6.3 Loss function

The loss function measures the difference between the predicted output and the actual output, and the aim is to minimize this difference during training. We trained the model using categorical cross-entropy to ensure optimal performance. It is a commonly used loss function for multi-classes classification problems, such as sequence generation, where the number of unique characters presents the classes. Once the SoftMax activation function gives the predicted distribution output, the categorical cross-entropy loss function calculates the difference between the predicted probability distribution and the actual one, where the actual distribution is represented as a one-hot encoded vector. Throughout the training, the model predicts the character token for each input token in the sequence. At each step the loss L is calculated as the categorical cross-entropy between the predicted and actual next token through this formula: At each step, the loss *L* is calculated as the categorical cross-entropy between the predicted and actual next token through this formula:

$$L\left(\hat{y},y\right)=-\sum_{i}y_{i}\;\text{log}\left({\hat{y}}_{i}\right)$$ (3.2)

In Eq (3.2), where *y* is the actual element and $\hat{y}$ is the predicted element for class *i*. For better understanding, here is an example: Suppose the actual distribution is [0, 0, 1, 0] while the predicted distribution is [0.14, 0.54, 0.13, 0.18]. Therefore, the cross-entropy loss function would be:

$$\begin{array}{cc} L & =-[(0\cdot \text{log}(0.14))+(0\cdot \text{log}(0.54))\\ & \;+(1\cdot \text{log}(0.13))+(0\cdot \text{log}(0.18))] \end{array}$$ (3.3)

The result will be approximately 2.04, which is relatively high, indicating that the predicted distribution does not match the actual distribution well. A smaller cross-entropy loss suggests a better fit between the expected and actual distributions. By minimizing this difference, the model can learn to generate molecules that are similar to the ones in the training set.

#### 3.6.4 Optimizer

The optimizer adjusts the model parameters during training depending on the gradients of the loss function. In our model, we employed the Adam optimizer to fine-tune the model’s parameters [29] and improve the performance. It minimizes the cross-entropy loss function between the prediction and the ground-truth value. It is a variant of stochastic gradient descent that adapts the learning rate for each parameter. It calculates the gradient of the loss function with respect to each parameter and uses this gradient to update the values of the parameters in the direction that minimizes the loss. The adaptive learning rate ensures that the model converges faster and more reliably to the optimal solution.

### 3.7 Training strategy

**Fig 10:**
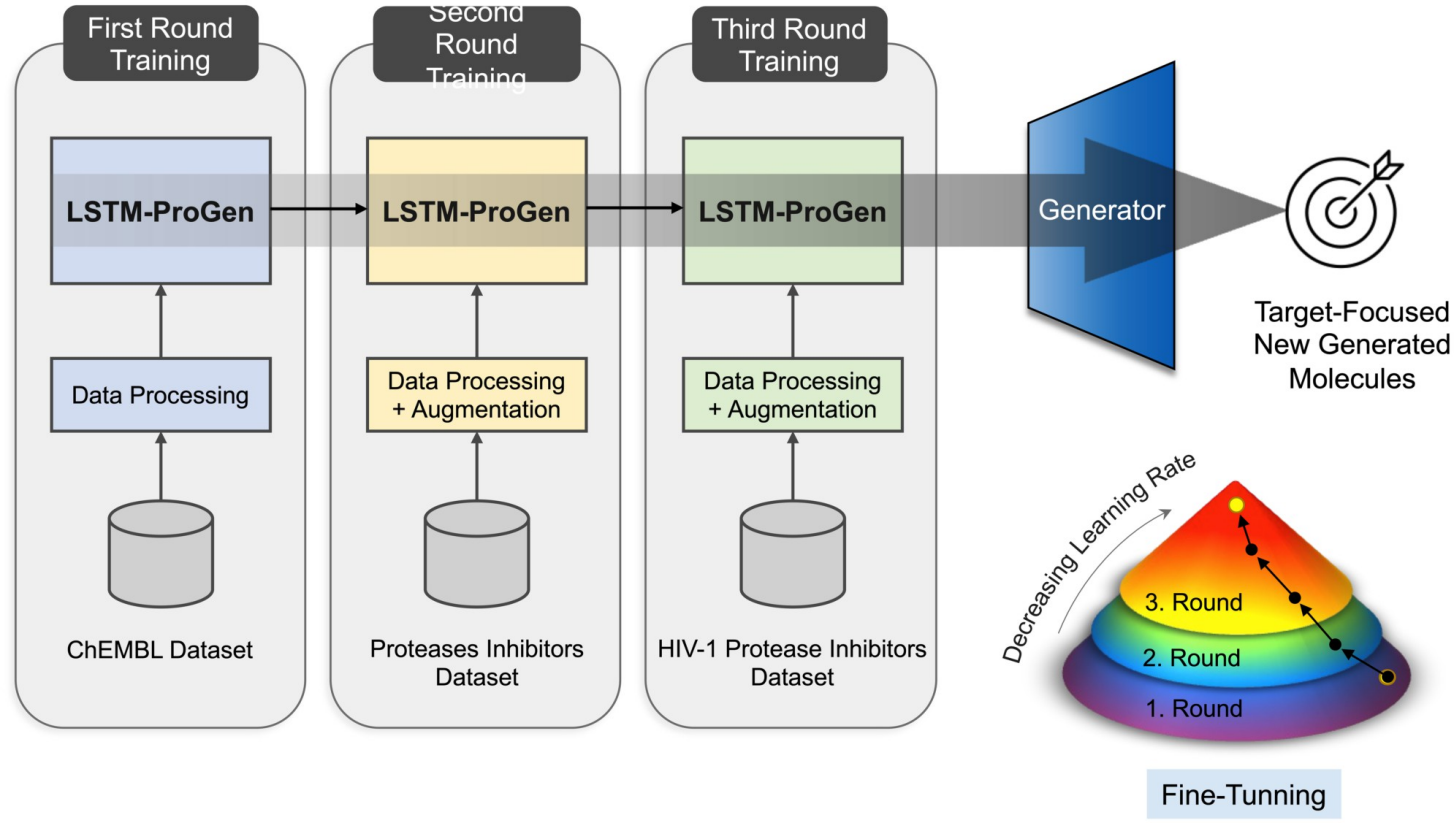
Overview of the training strategy we followed to train our models. The training strategy includes three rounds: the first round with the ChEMBL 14K drug dataset, the second round with the protease inhibitors dataset, and finally the third round with the HIV-1 protease inhibitors. At the right bottom corner, the figure simply showed the employed fine-tuning process to guide the generation process; in each step, the learning rate decreased to make the molecules focus on the target and generate similar molecules to those in the training sets. It starts in purple, where the model learns to generate drug- like molecules in general, then narrows up to focus only on protease inhibitors, and finally, in the last round, in red, focuses on HIV-1 protease inhibitors.

In this study, we trained the defined model with the previously mentioned datasets following a novel strategy Fig 10 for the purpose of guiding the generation process to focus on the target. It consists of three rounds. Firstly, the model was wormed up with the (14K drug) dataset, followed by the protease inhibitors dataset in the second round, and finally with the HIV-1 protease inhibitors dataset in the third round. Each time, we decreased the learning rate and assessed the model’s performance to ensure its accuracy was improving. In this way, we were able to improve the quality of the generated molecules by biasing the generation to focus on the last added dataset and generate new active molecules against the HIV-1 protease, and quite similar to those in the last training sets.

### 3.8 Molecule filtering

The following processes are used to identify the best de novo new candidate molecules produced by the LSTM-ProGen model for targeting the HIV-1 protease, based on the partition coefficient of a solute between octanol and water (LogP), molecular weight (MW), and ring types collected from the analysis of the HIV-1 protease known inhibitors dataset. Compounds that don’t share similar features are eliminated based on the calculated Tanimoto similarity scores estimated using Morgan fingerprints between the newly generated molecules and those in the original set of HIV-1 protease inhibitors, the top 10 molecules were selected with the greatest Tanimoto similarity scores.

### 3.9 Molecular docking

Molecular docking is a computational technique that predicts the binding orientation and affinity of a small molecule ligand to a protein target. It is a crucial step in drug discovery and development, as it can help identify potential lead compounds that can bind to the target protein with high affinity and specificity. The molecules that remained after the filtering operations were analyzed via molecular docking. First, the molecule library was created using Open-Babel software [30]. For the docking study, the crystal structures of HIV-1 protease (PDB code “4HLA” [31] and PDB code “3S45” [32]) were prepared by Discovery Studio 2021 software [33]. Missing hydrogen atoms were added, and water molecules were removed. The physical condition of pH was set as 7.4 ± 1.0 for atom typing. The binding site coordinates of 4HLA were defined as center x = 8.16, center y = -16.04, and center z = 0.010, and 3S45 were defined as center x = 20.51, center y = 11.30, center z = 18.08, by detection with the same software and cross-checked with the data published in the literature. PyRx software [34] was used to find the best binding poses for each ligand within both used structures. It is a free and open-source software package, that combines several open-source software, including AutoDock [35], AutoDock Vina [36], and Open-Babel [30] that provides a user-friendly graphical interface for performing molecular docking studies of large compound libraries against protein targets. After The AMBER force field [37] and AutoDock Vina [36] search algorithm utilized in PyRx for the docking simulation, the results were analyzed and visualized with PyMOL [38] and Discovery Studio software [33]. In summary, this part outlines the methodology employed to achieve the study’s goal. A comprehensive overview of the process, including the utilization of the ChEMBL database for input molecules and the extraction of relevant sub-datasets, and the idea behind the exploration of the HIV protease known inhibitor dataset, revealed important features for further analysis. Additionally, a data augmentation method was applied to enhance dataset diversity and improve the model’s performance. The data processing technique was thoroughly explained, ensuring transparency and reproducibility. The part also discussed the model structure and training strategy. A filtration process was performed on the resulting generated molecules to minimize their size, select the most relevant molecules with high hopes of having inhibitory activity against the target, and decrease the computational cost that the molecular docking study requires. All these elements together enhance the credibility and impact of the study’s findings.

## 4 Experimental part

### 4.1 Test environment

In this work, the datasets were processed using the Tesla K80 GPU, which Google Colab provided with 12 GB of GDDR5 RAM. LSTM-ProGen proposed molecular models were trained using this GPU to ensure optimal performance without the need for costly hardware resources. It meets the computing needs of the training and data processing tasks. When the training was completed, the models were stored on disk for future use. Once the training operation was completed, the last round of training was saved on the disk. The generator function is executed on the HP ProBook 455 G4 computer, which has an AMD A10–9600P RADEON R5 processor with 10 computer cores, 8.00 GB of installed RAM, and a 64-bit operating system running Windows 10 Pro (20H2). In this work, a harmonic ensemble of libraries gathered within the execution of our models, by leveraging the capabilities of Pandas, NumPy, Selfies, Scikit-learn, Seaborn, RDkit, Matplotlib, and TensorFlow libraries. These libraries acted as trusted companions, offering practical solutions, and improving our workflow, propelling us closer to our goals. The model architecture was defined using the sequential model type provided by the TensorFlow library [39], giving optimum training flexibility. Each training iteration used the well-known Adam optimizer and consisted of 64 batches and 20 epochs. A molecular docking study was performed on the final filtered molecular library. The docking study employed the same gear as previously described. PyRx software was the main software utilized for this task together with different software such as Discovery Studio and OpenBabel to achieve the goal. PyMOL software [38] was also used to visualize the results.

### 4.2 Testing plan

For further examination, we tested two models with a slight variation in the data processing part for the purpose of identifying the most effective strategy for the best performance of our model. The two models have the same multi-layer architecture and are gradually trained using the same three-round technique as described before Table 2. Through the implementation of these two models (Alpha and Beta), we highlight the significant impact of specific factors on the model outcomes. We evaluated the significance of maintaining a fixed maximum sequence length that we padded all the sequences to as a data processing part and a common token dictionary used to turn the molecules into vectors. The variation in the number of input molecules across different rounds in both Alpha and Beta models, as depicted in Table 2, arises from our meticulous curation process. Initially, the “14K drugs” from ChEMBL database containing approximately 14,000 authorized small molecules with drug-like properties was utilized. From this, 9,480 molecules with characteristics akin to well-known HIV-1 protease inhibitors were selected. Subsequently, another dataset focusing on protease inhibitors for various viruses with mechanisms comparable to HIV-1 protease inhibition was created, resulting in 391 molecules after filtering. Additionally, a set of 17 approved HIV-1 protease inhibitors was extracted from the same source. Due to the relatively small size of the second and third datasets for model training, we employed the “SMILES Enumeration” technique to increase the number of samples in each of them. This augmentation expanded the second dataset to 3,147 molecules and the third to 2,600 molecules. These datasets were used for training the Alpha and Beta models. However, to assess the impact of training the Beta model with molecules of a fixed length, we filtered the second and third datasets to include only molecules shorter than 100 atoms. As a result, the datasets for Beta comprised 3,147 and 1,717 molecules, respectively. Our analysis revealed a significant improvement in stabilizing the learning process, enabling the Beta model to perform admirably and generate valid molecules from Alpha, as detailed in the results section. The varying lengths used in the first model throughout each training cycle increased the complexity of the molecule production process. Consequently, the quality of the molecules produced decreased, and many of them proved to be invalid.

**Table 2:** Comparison of model specifications for two LSTM-based models.

Model Specifications	Alpha			Beta		
	Round 1	Round 2	Round 3	Round 1	Round 2	Round 3
Number of input molecules	9480	3819	2600	9480	3147	1717
Number of unique characters	84	57	56	86	86	86
Max length	95	462	108	100	100	100
Optimizer	Adam	Adam	Adam	Adam	Adam	Adam
Learning rate	0.005	0.005	0.003	0.005	0.004	0.003
Epochs	20	20	20	20	30	30
Batch size	64	64	64	64	64	64

The configuration parameters, including the number of input molecules, unique characters, sequence length, optimizer choice, learning rate, and training parameters, for Alpha and Beta across multiple evaluation rounds.

The Beta model ensured a more regulated and organized generating process by enforcing a set maximum sequence length. This limitation allowed for more control over the output, boosting the possibility of regularly creating legitimate and high-quality molecules. Furthermore, the constant token dictionary employed throughout all training rounds provided a consistent foundation for the model’s knowledge and representation of molecules. As a result of these modifications, the second model greatly outperformed the first model. The stated maximum sequence length and constant token dictionary made molecule formation more reliable and genuine. This circumvented the constraints indicated in the original model, allowing for the synthesis of higher-quality and more valuable molecules. Moreover, the performance of LSTM-ProGen models in designing de novo molecules was evaluated using well-known metrics with conducting a comparative analysis to address the limitation of each proposed model and indicate the impact of the length and number of tokens factors on the learning process. For a better understanding of the chemical space occupied by the de novo molecules generated by the Beta model that was considered as a baseline model for this study, we used the common powerful technique of principal components analysis (PCA) in conjunction with 2D and 3D visualization. We were able to translate the high-dimensional molecular data into a lower-dimensional representation while keeping the fundamental structural properties by using PCA. Because of the decrease in dimensionality, we were able to explore and understand the complicated chemical space in a more intuitive and interpretable manner. In the 2D visualization, the created de novo molecules were projected onto a two-dimensional plane based on the dataset’s major components. Each point on the diagram represented a distinct molecule, with its position representing the overall structural features of the molecule. We learned about the similarity and variety of the created compounds with respect to known chemical compounds by comparing the distribution and clustering patterns of the de novo molecules with those of training molecules. We also expanded the technique to 3D visualization to improve our grasp of the chemical space.

We acquired a more thorough depiction of the structural changes and connections between the molecules by integrating an extra dimension. The resulting 3D plot showed the de novo compounds in a visually appealing manner, allowing for a more in-depth investigation of their structural variety and possible chemical attributes. We were able to show areas inside the chemical space filled by the de novo molecules by rotating and studying the 3D map from various perspectives. This comparison of de novo molecules with real molecules allowed us to evaluate the novelty and differentiation of the generated compounds. We were able to determine if the produced molecules were located in the same spots known for compounds or explored uncommon chemical space. This evidence provides important insights into the possibility of developing novel chemical structures and providing prospective leads for drug research by the proposed approach of LSTM-ProGen. Therefore, the combination of PCA-based 2D and 3D visualization methods produced a visually appealing and educational representation of the chemical space explored by de novo molecules. It allowed us to investigate their distribution, diversity, and likeness to real molecules, giving valuable information about the quality and potential applications of the generated compounds. In addition to examining the chemical space of the de novo molecules, we used a molecular docking experiment to ensure their target-specific activity. This method revealed important information about the possible interactions and binding affinities of the generated molecules by LSTM-ProGen with our specific target proteins. Molecular docking was used to simulate the binding process between the de novo molecules and the HIV-1 protease target proteins. The compounds were rated for binding affinity, which indicates their potential activity and efficacy as target protein ligands. Furthermore, two structures were employed in the investigation, and they are available on the Protein Data Bank (PDB) to guarantee the validity of the findings. We were able to discover promising compounds with high binding affinities and strong interactions with the target proteins through this experiment investigation. These molecules might benefit from further experimental validation or optimization in drug development efforts. By integrating the results of the molecular docking analysis with the chemical space exploration, we were able to obtain a complete picture of the potential biological activity of the generated compounds. We were able to prioritize and choose compounds with desirable structural features as well as favorable binding behaviors by benefiting from the combination of computational predictions and visualization-based research. The target-specific activity ranking based on molecular docking added an important dimension to the evaluation of the de novo molecules. It bridged the gap between their structural features and likely biological activities, allowing for a more thorough evaluation of their overall drug-like properties and therapeutic implications. As we wrap off this session, we look back on our rigorous testing method, which includes the evaluation of two versions of LSTM-ProGen. Minor adjustments were made in the data processing stage, which is critical to ensuring high-level performance.

Our main objective was to discover the best-performing model for creating biased chemicals. In our quest for excellence, we did a further examination of the major impact of various methodologies. We explored the intricacies of various tokenization techniques and evaluated different sequence lengths, extensively assessing their influence on model performance. We were able to discover the ideal arrangement for moving us closer to our goal. However, our investigation did not end with a performance evaluation. We descended into the depths of chemical space, eager to ascertain the authenticity and potential activity of the generated molecules. The principal component analysis (PCA) was performed in 2D and 3D visualizations way. Finally, we sought to validate their putative action by putting these de novo molecules in the intriguing realm of molecular docking. We were able to determine their ability to interact with and inhibit the target protein of our highest interest using this strategy. We used molecular docking to analyze the binding affinities and interactions of the produced compounds with the target protein, indicating their potential as promising candidates in our hunt for selective inhibition.

## 5 Results and discussion

This section focuses on the outcomes of using the Alpha and Beta LSTM-ProGen models. The two models’ performances were compared and well discussed for the task of generating new HIV-1 protease inhibitor candidates. Novel compounds were produced by the LSTM-ProGen architecture that had not been found before in the training dataset. Additionally, the results of the molecular docking experiments are well-reported.

**Fig 11:**
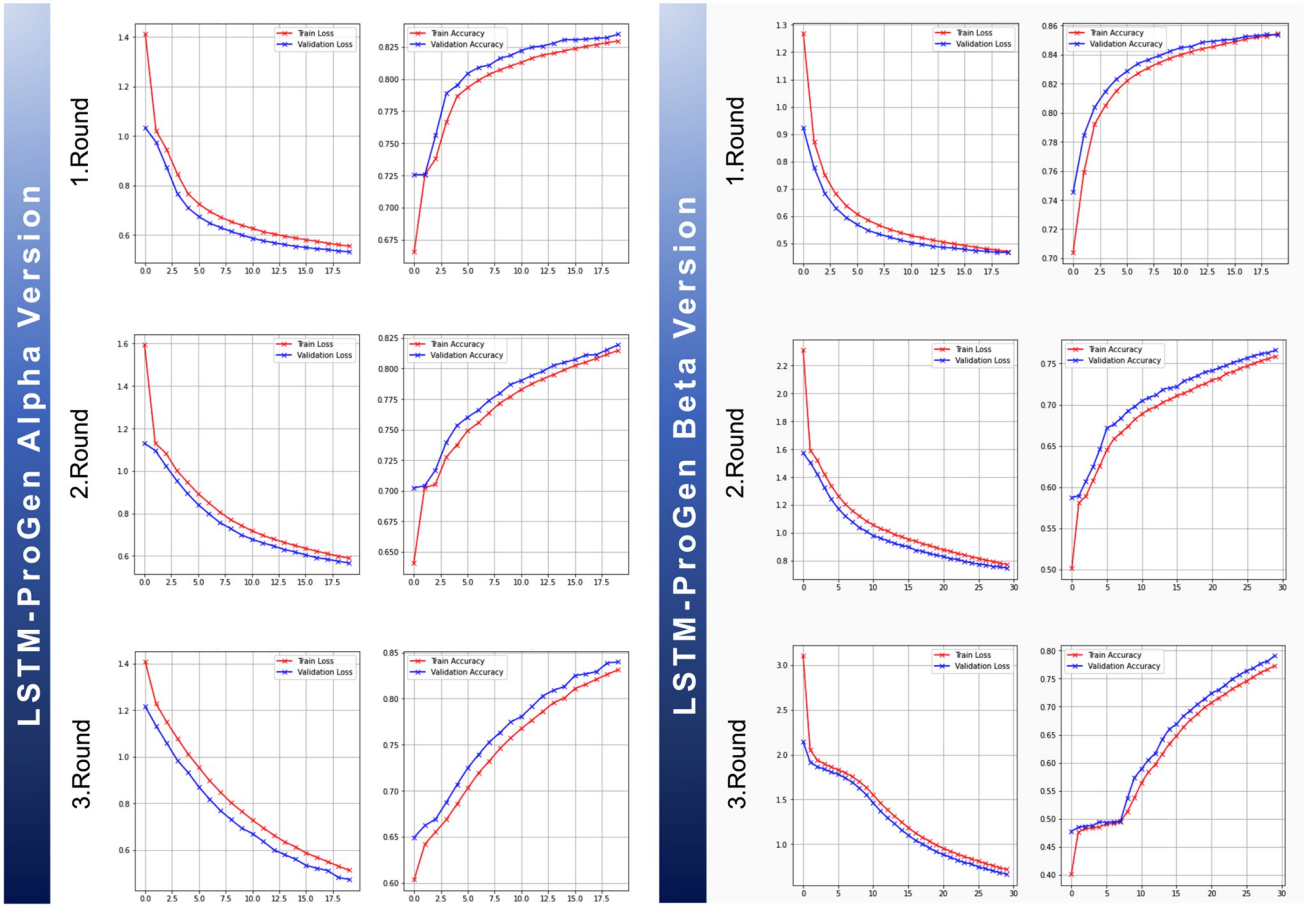
Alpha and Beta models performance trends. Performance improvement across three training rounds for both the Alpha and Beta LSTM-ProGen models. For each scenario, plots of accuracy and loss against the number of epochs demonstrated a consistent improvement in model performance.

Before diving into the details of our discussion, let’s establish a solid foundation by understanding the primary objective of any generative model. These exceptional models employ their talent to create new samples that are comparable to the training instances while also recognizing the distribution of the hidden features in the training examples, sparking a creative symphony of possibilities. While undergoing the process of training, one way to evaluate the model’s performance is the training accuracy metric, which measures how well the model recreates the training data during the training process. As the model progresses in learning, it is expected to improve its capability to sample the training data, resulting in a rising training accuracy curve, while the validation accuracy of a generative model is another one that often depends on how well the model generalizes untested data. Fig 11 shows the full training progress over the three training rounds of the two variant models (Alpha and Beta).

The Alpha model demonstrated consistent, smooth training curves, displaying a relatively balanced relationship between loss and accuracy over all training iterations. Similarly, the Beta model achieved similar performance across the three training rounds, with a slight variation in the first round, indicating a sign of overfitting tendencies towards the end of training. It is presented by the intersection of the training and validation accuracy curves during the last epochs. It might have happened because the model started to focus on memorizing the training data or capturing specific features. It mostly suggests a potential likelihood for the model to become less effective at generalizing to unseen data. Though it is worth noting that this situation did not continue in the next two rounds of training, revealing that it was not a serious worry that required immediate action, an early stopping regularization procedure with patience of 3 was applied in the subsequent rounds. This preventative step helps to keep the worst-case scenario and any prospective difficulties from negatively impacting performance during the training process.

In general, both models Alpha and Beta, performed similarly across all training rounds, and the scores of performance metrics reported in Tables 3 and 4 clearly show that both models scored quite close to one other. However, upon further examination of the generated molecules utilizing both models, despite the slightly inferior overall performance of the Beta model, it provided a notable capability to generate molecules with higher validity scores compared to the Alpha model. Training the Alpha model on different lengths with different numbers of unique characters for encoding molecules probably caused some confusion throughout the training process, resulting in low-validity molecules created using this model. Conversely, training the Beta model with a consistent length and the same set of unique characters for the encoding process during all the training rounds increased its ability to learn rules and generate valid molecules better than the Alpha model. Based on this, these two elements are essential for guaranteeing stability throughout training and producing reliable molecules.

**Table 3:** Comparison of Alpha and Beta performance across multiple evaluation rounds.

Metrics	Alpha			Beta		
	1st R	2nd R	3rd R	1st R	2nd R	3rd R
Acc	85.34%	76.61%	79.07%	83.49%	81.92%	83.97%
TP	25759	11259	8129	25630	16207	1557
TN	113046	24999	8459	103551	69661	16053
FP	405	124	46	459	343	0
FN	189	97	21	157	117	7

**Table 4:** Performance metrics.

	Accuracy	Precision	Recall	F1-score
Alpha	0.99	1.0	0.995	0.997
Beta	0.995	0.994	0.997	0.995

Considering this significant observation, the Beta model was chosen as the benchmark model to forward the research into developing de novo compounds that target the HIV-1 protease. With the Beta model exhibits exceptional promise and the ability to provide big outcomes. Furthermore, it is regarded as a beginning point for future advancements, since it opens the door to additional work to generalize the used framework to be robust and effective in the development of molecular modeling.

**Fig 12:**
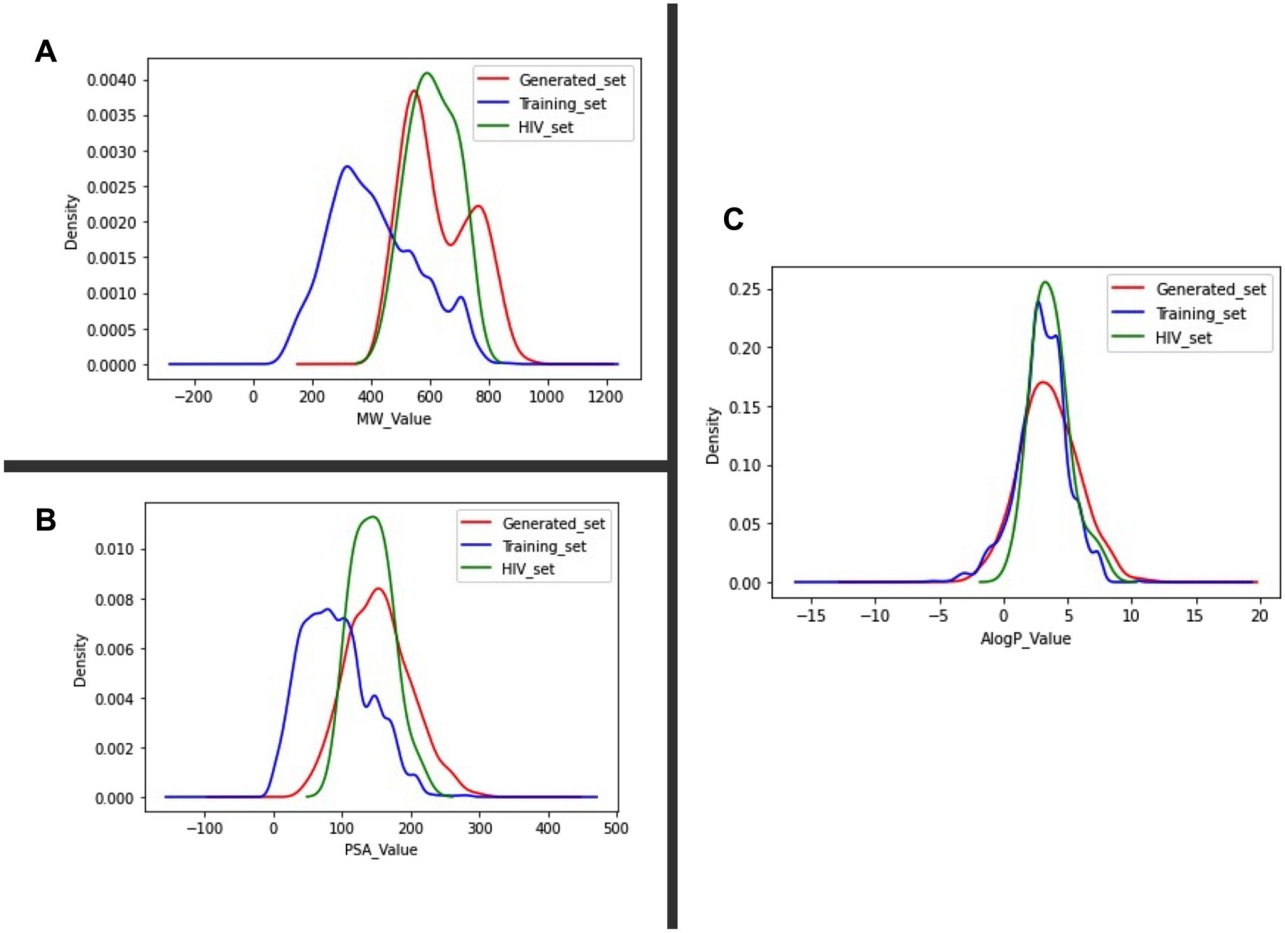
Analysis of chemical descriptors distribution among datasets. Kernal density similarity plots for comparative analysis of the distribution of three chemical descriptors, including molecular weight (MW), octanol-water partition coefficient (AlogP), and polar surface area (PSA), among the three datasets, training (blue), HIV inhibitors (green), and the generated molecules by LSTM-ProGen (red) datasets. (A) displays that the molecular weight range varies slightly between the mentioned datasets, but the newly generated set adequately covers the same ranges as the HIV inhibitors dataset. (B) shows the polar surface area ranges are very similar, with only a slight upward trend observed in the HIV dataset. (C) highlights the similarity in the range of AlogP values across all three sets.

Utilizing the Beta model, approximately 3000 novel molecules were generated, which were entirely distinct from those present in the training set. Scaffold variety measures the diversity of key molecular scaffolds in generated compounds [40]. It provides higher-level insights into the range of generated chemical structures. The 3000-molecule sample contained 1760 distinct core scaffolds. It is an important clue that the dataset’s newly produced molecules have a wide range of diversity. In addition, the novelty score calculated the percentage of produced molecules that differed from the molecules in the training dataset. It evaluates the model’s ability to create previously undiscovered chemicals. It was 100%, suggesting that all newly generated molecules are original and unique, with no duplicates or similarities with the training samples. Moreover, to gain insights into the physicochemical properties of these newly generated molecules, the density plots of three descriptors, namely molecular weight, octanol/water partition coefficients (AlogP), and polar surface area (PSA), were analyzed in Fig 12. The plots revealed significant similarities in the ranges of these descriptors among the generated molecules, HIV inhibitors, and the training dataset of ChEMBL molecules.

**Fig 13:**
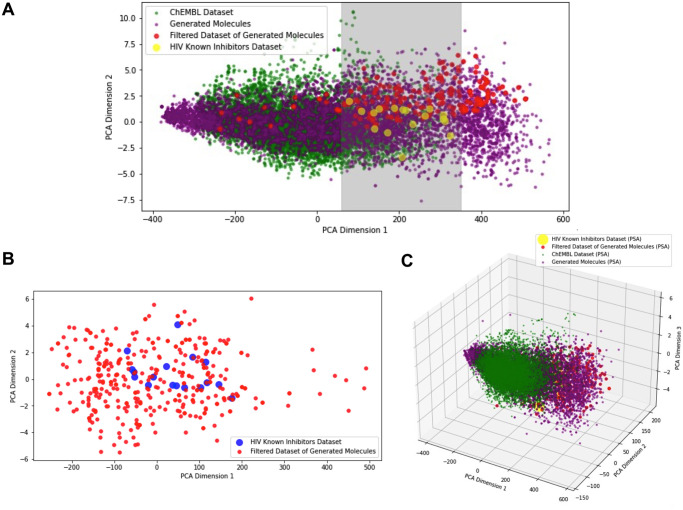
Exploring chemical space. Mapping the chemical space through principal component analysis (PCA) among the LSTM-ProGen model- generated molecules, the training dataset, and the HIV known inhibitors dataset. (A) demonstrates physicochemical properties such as the molecular weight and octanol-water partition coefficient (LogP) in three datasets. (B) HIV inhibitors and the new filtered molecules aligned. (C) Beyond 2D: A Multi- dimensional Illustration of Molecular Weight, Octanol-Water Partition Coefficient (LogP), and Polar Surface Area (PSA) in the three datasets.

Afterward, filters derived from known HIV protease inhibitors were applied to reduce the size of the generated set and focus on molecules with probably high activity against HIV-1 protease. This filtration process resulted in a subset of 360 molecules. To further investigate the chemical space covered by the de novo molecules, a principal component analysis (PCA) was conducted. Fig 13 showcases the relationship between the entire generated molecules dataset, all the HIV inhibitors, and the entire training dataset of ChEMBL with the high-spread diversity features. This analysis aimed to explore the proximity of the generated molecules to the HIV inhibitors dataset, which is of particular interest in this study. De novo molecules of the LSTM-ProGen model and Chembl molecules are mostly spread out over the same space. The red points indicate the filtered dataset which is expected to cover the near space that the real HIV inhibitors in yellow cover. This ended up with fair affinities in the molecular docking results as provided in the next section.

This reduction of the number of samples was necessary to minimize resources and the computational cost associated with the subsequent molecular docking study. Through a thoughtfully curated expert-led selection process, a subset of 76 compounds was randomly chosen from the pool of filtered de novo molecules. In this way, we ensure the high ability to be active and lower the computational cost required for the docking study.

### 5.1 Molecular docking results

The molecules that remained after the filtering operations were tested via molecular docking. Strong evidence was determined to support the significant potential of several of our tested de novo compounds to be active against HIV-1 protease with inhibitory activities. These findings are an essential first step in unraveling the complexity of drug-target interactions and paving the way for future therapeutic development advances.

**Fig 14:**
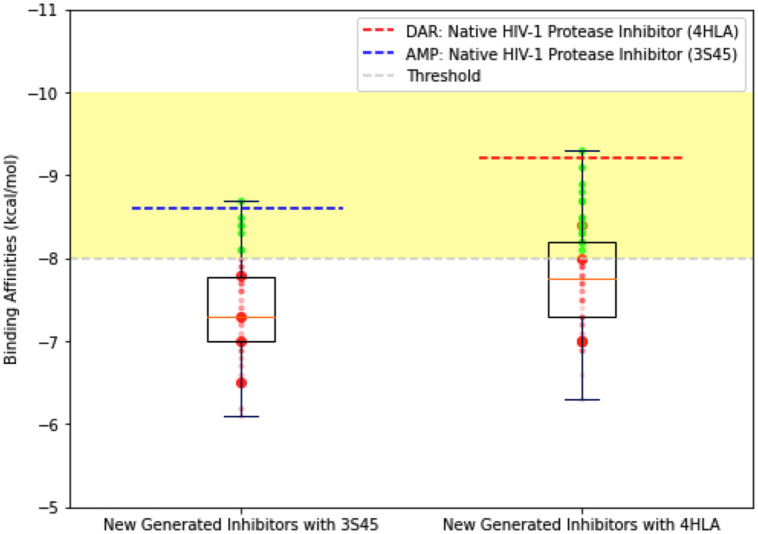
Docking analysis. Score box plots displaying the binding free energies measured in the docking analysis of dene novo molecules generated by the LSTM-ProGen model with two different HIV- 1 protease structures (4HLA and 3S45) provided on the protein data bank. The scores of both the native ligand (Darunavir) in the utilized HIV protease complex structure are shown by the red dashed line, and another known inhibitor (Amprenavir) is shown by the blue dashed line. The green points indicate the newly generated compounds with the potential to inhibit the HIV-1 protease.

In our application, a comprehensive cross-evaluation method was implemented to validate the docking process and ensure the reliability of our protocol. The goal was to evaluate not only the activity of our de novo molecules but also the efficacy of well-established and approved HIV protease inhibitors to confirm the validity of our protocol. The docking results of both the known inhibitors and our novel molecules were compared. To initiate the cross-evaluation, a docking study was performed on the native HIV protease inhibitors, utilizing the structures observed in the Protein Data Bank “PDB”. DAR was docked with the 4HLA structure, while AMP was docked with the 3S45 structure. The docking scores of DAR and AMP were carefully determined, considering their interactions, and fit within the binding regions of the target protein. Their scores were -9.2 and -8.6, respectively. These inhibitors served as reference points due to their well-documented affinities and binding abilities. Subsequently, our new compounds underwent the same docking technique, and their docking scores were compared to those of the native inhibitors mentioned above. The activity threshold was set at -8, indicating that any compounds scoring lower than this value had the potential to significantly inhibit the target protein. The docking scores are presented in Table 5. By ensuring that several of the tested de novo molecules achieved docking scores comparable to or higher than those of DAR and AMP in the case of Mol 92 with 4HLA and No 145 with 3S45. In this way, the credibility and efficacy of our protocol were established. This cross-evaluation method enabled the validation of the docking process and the verification that our molecules exhibited similar binding affinities and interactions within the active site of the target protein. Fig 14 shows the de novo molecules that were able to achieve affinity scores smaller than -8, highlighted in green. Furthermore, two of them surpassed the native inhibitors of the two utilized structures, DAR in 4HLA and AMP in 3S45.

**Table 5:** The newly generated molecules with the highest binding affinity scores in both structures of HIV-1 protease (4HLA and 3S45).

4HLA PDB Structure		3S45 PDB Structure	
Ligand ID	Binding Affinity	Ligand ID	Binding Affinity
Mol 092	-9.3	No 145	-8.7
Mol 154	-9.1	No 042	-8.5
Mol 042	-8.9	No 127	-8.4
Mol 127	-8.8	No 285	-8.4
Mol 145	-8.7	No 245	-8.3
Mol 195	-8.7	No 018	-8.1
Mol 211	-8.7	No 195	-8.1
Mol 181	-8.5	No 154	-8
Mol 245	-8.5	No 295	-8

**Fig 15:**
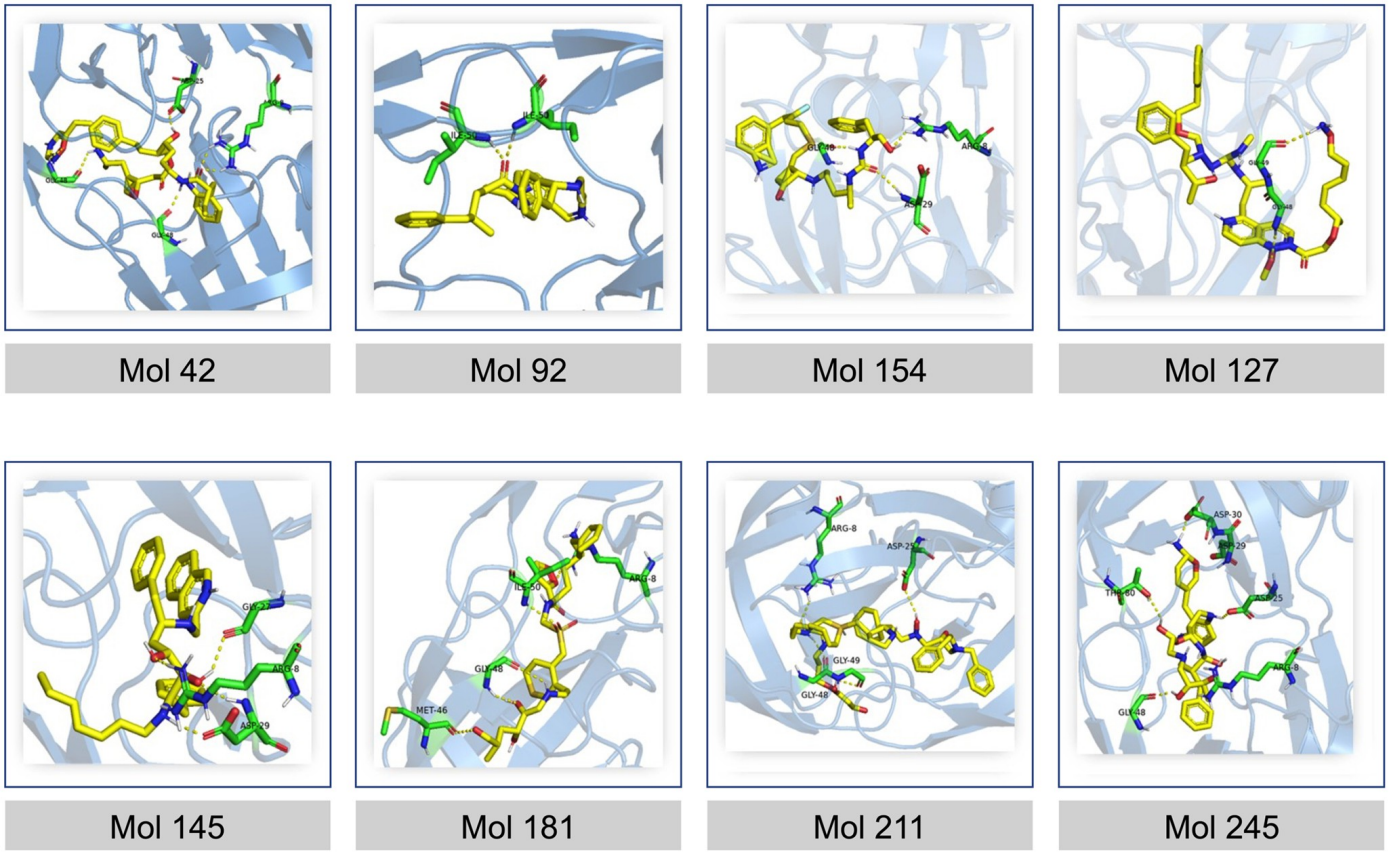
Visualization of docking study results. HIV-1 protease complex structure with the Darunavir inhibitor (PDB id: “4HLA”); visualization of the best- predicated poses of the molecular docking study of the de novo generated inhibitors of HIV-1 protease in its active site. The PyMOL program was used to create these figures.

**Fig 16:**
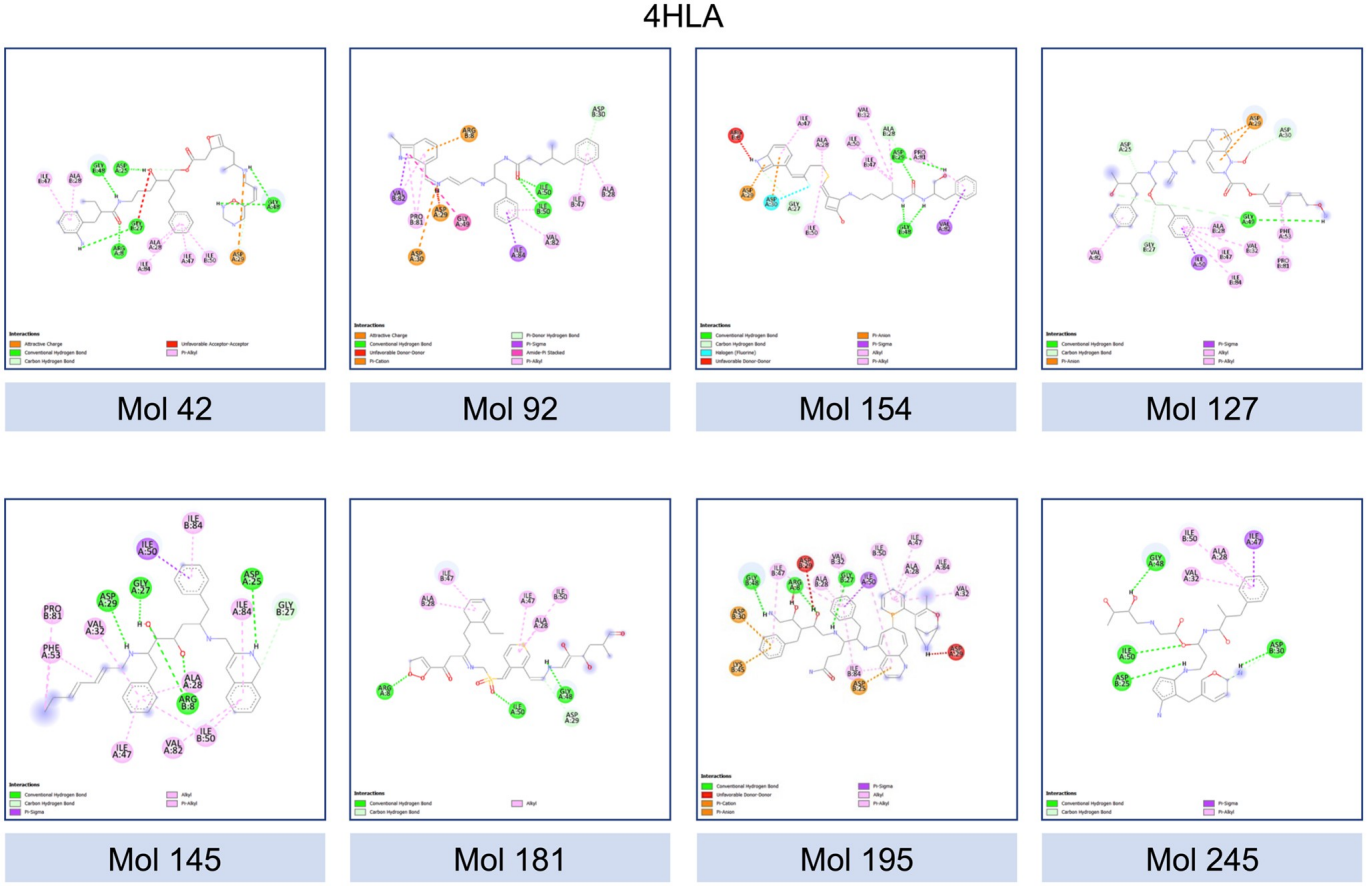
Exploring receptor-ligand interactions. Analysis of docking study results using Discovery Studio software, revealing crucial receptor-ligand binding interactions.

For the visualization and analysis of the resulting complexes, advanced molecular visualization tools such as PyMOL were employed. The three-dimensional structures of the complexes were rendered, enabling the examination of the spatial arrangement and interactions between the molecules and the target protein Fig 15. Additionally, Discovery Studio, a comprehensive software suite, was utilized to explore the interactions in the complexes Fig 16. This powerful tool facilitated a thorough investigation of the properties and characteristics of the docking complexes, allowing for a deeper understanding of the molecular interactions, and aiding in the identification of key binding residues. Through this meticulous cross-evaluation method, the reliability of our docking protocol was validated by confirming that our novel molecules exhibited comparable affinities to the well-established and approved inhibitors. This approach not only strengthened the credibility of our results but also provided valuable insights for the identification and development of potential HIV protease inhibitors with promising activity against the target protein.

To sum up, a set of 76 molecules was selected from the filtered set to test their inhibitory activity against the target protein through molecular docking studies. A final set of 12 molecules demonstrated significant potential as inhibitors for HIV-1 protease, thus offering potential avenues for the treatment of AIDS/HIV. These selected molecules exhibited favorable binding affinity scores, indicating their ability to interact effectively with the target protein and potentially disrupt its function. However, it is important to note that out of the initial pool of 76 molecules, only 12 showed inhibitory activity in the docking experiments. This observation highlights the need for further optimization and refinement of the selected molecules, as well as improvements to the proposed generation model. Enhancements in these areas can lead to the development of more effective inhibitors with enhanced binding affinities and improved therapeutic potential. The identification of these 12 molecules with promising inhibitory activity through molecular docking represents an important step in the drug discovery process for HIV-1 protease inhibitors.

It is important to acknowledge that this study is based on a computational approach; therefore, the feasibility of synthesizing the suggested molecules has not been examined. Additional experimental validation and evaluation are crucial to validate their inhibitory activity and assess their potential for further development as therapeutic agents.

## 6 Conclusions and future work

LSTM-ProGen is an automated approach for designing target-specific therapeutic candidate molecules. Combining the power of LSTM-based modeling with the robustness of Selfies molecular language, our model presented a remarkable performance in producing HIV-1 protease inhibitor candidates, demonstrating the efficacy of employing LSTM in this endeavor. One of LSTM-ProGen’s primary strengths is its high-generation efficiency and capacity. The model was able to generate de novo compounds with similar physicochemical properties to known HIV-1 protease protein inhibitors by efficiently learning from different datasets. Various physicochemical metrics, such as polar surface area (PSA), molecular weight, and the logarithm of the partition coefficient (logP), revealed that the new molecules were within the same drug-likeness limits as the known HIV-1 protease inhibitors. Further computational studies were conducted to assess the target-specific features of the de novo molecules generated by LSTM-ProGen against the target protein. The results indicate a high potential for targeting HIV-1 protease, a key enzyme in the HIV replication cycle. Through the molecular docking study, the de novo compounds exhibited binding affinities and interactions comparable to current HIV-1 protease inhibitors, suggesting their potential to be optimized to reduce viral replication. The findings show LSTM-ProGen’s ability to rapidly explore chemical space and design novel molecules with desirable properties for specific protein targets. The model’s ability to generate molecules with properties similar to those of known inhibitors provides a chance for faster drug discovery and development processes. Although LSTM-ProGen faced difficulties in effectively capturing the rules governing ring systems, the LSTM-ProGen approach remains a valuable tool for streamlining the discovery and development of new drugs for druggable targets, as it helps to reduce the time-consuming and costly aspects associated with traditional hit-to-lead optimization. While LSTM-ProGen demonstrated promising results, additional experimental validation tests are required to confirm the efficacy and safety of the presented molecules. In vitro and in vivo studies, as well as rigorous toxicity assessments, are critical steps in determining the viability of these de novo molecules as drug candidates.

As future work plan is concerned, it can be easily realized that the advancements in deep learning (DL) and machine learning (ML) approaches have made a significant impact on the field of drug discovery. These approaches have shown promising results with the goal of speeding up the identification and design of novel drug candidates. LSTM-ProGen stands as evidence of the importance of DL and ML in this domain. However, certain areas require more research and improvement to continue to enhance the outcomes of such models. One of the most important critical challenges that we plan to address is the development of a technique to improve learning during the training process to enable the production of molecules with ring structures similar to those found in approved drugs to date. LSTM-ProGen had suffered from the learning of the ring systems, and this is revealed in the resulting molecules. One potential option could be to train LSTM-ProGen on larger and more diverse datasets. By exposing the model to a wide range of molecular structures, including those with ring systems, it may learn to generate molecules more closely resembling approved drugs. However, this approach may necessitate significant computational resources as well as careful curation of the training data. Another possible choice is fragment-based training, which provides a novel and innovative solution to the problem of generating molecules with the correct ring systems. We can capture the rich structural diversity found in existing drugs and medicinal compounds by training a separate model on a dataset consisting of diverse ring fragments. This fragment-focused model learns the intricate relationships between various fragments, such as connectivity and spatial arrangements. As a result, it gains a thorough understanding of the essential characteristics and properties of ring structures, enhancing its ability to generate molecules with appropriate ring structures that are similar to those in the approved drugs to date. Afterward, we can transfer learning to the main model implemented for a certain subject; following this way, we can ensure to generate molecules with the desired properties for the subject of the study with proper ring structures. In addition to these structural considerations, and leaving aside the computational cost, reinforcement learning can be a valuable tool for guiding the generation process. Through reinforcement learning, the model can adapt its generation strategy based on the feedback received from the evaluation process. This method allows the model to prioritize specific properties during the generation process, such as efficacy, safety, or target specificity, resulting in the discovery of molecules with improved pharmacological profiles. To sum up, DL and ML hold great promise for revolutionizing drug discovery and bringing tremendous hope in positively impacting patients’ lives. However, further advancements are necessary to fill the existing gaps and accelerate this process. By addressing the challenges associated with generating molecules, we can enhance the performance and utility of AI-based models in the field. This pursuit of improvement is essential to our collective mission to save lives, support medicine, and enhance the well-being of patients worldwide.

## Supporting information

S1 Checklist PLOS ONE clinical studies checklist. (PDF)
